# Recombination hotspots attenuate the coupled ATPase and translocase activities of an AddAB-type helicase–nuclease

**DOI:** 10.1093/nar/gku188

**Published:** 2014-03-15

**Authors:** Neville S. Gilhooly, Mark S. Dillingham

**Affiliations:** DNA-Protein Interactions Unit, Department of Biochemistry, School of Medical Sciences, University of Bristol, University Walk, Bristol BS8 1TD, UK

## Abstract

In all domains of life, the resection of double-stranded DNA breaks to form long 3′-ssDNA overhangs in preparation for recombinational repair is catalyzed by the coordinated activities of DNA helicases and nucleases. In bacterial cells, this resection reaction is modulated by the recombination hotspot sequence Chi. The Chi sequence is recognized *in cis* by translocating helicase–nuclease complexes such as the *Bacillus subtilis* AddAB complex. Binding of Chi to AddAB results in the attenuation of nuclease activity on the 3′-terminated strand, thereby promoting recombination. In this work, we used stopped-flow methods to monitor the coupling of adenosine triphosphate (ATP) hydrolysis and DNA translocation and how this is affected by Chi recognition. We show that in the absence of Chi sequences, AddAB translocates processively on DNA at ∼2000 bp s^−1^ and hydrolyses approximately 1 ATP molecule per base pair travelled. The recognition of recombination hotspots results in a sustained decrease in the translocation rate which is accompanied by a decrease in the ATP hydrolysis rate, such that the coupling between these activities and the net efficiency of DNA translocation is largely unchanged by Chi.

## INTRODUCTION

Double-stranded DNA breaks (DSBs) pose a serious threat to cells. Their illegitimate recombination or ligation can result in gross chromosomal rearrangements leading to genome instability, and failure to repair DSBs is potentially lethal (1). It is therefore essential that DSBs are repaired to ensure cell survival, and repair by homologous recombination offers a faithful mechanism by which genetic information lost at the break site can be salvaged (2,3).

In bacteria, the initiation of recombinational repair is catalyzed by helicase–nuclease enzymes (1,4,5). These are stable multi-protein complexes that possess the necessary helicase and sequence-regulated nuclease activities required to resect DNA ends (4). The AddAB complex is a model helicase–nuclease that initiates DSB repair in *Bacillus subtilis* (6). AddAB binds DNA ends extremely tightly and utilizes a single SF1A motor to translocate through and unwind the DNA duplex (7,8). Translocation of the 3′-strand by the AddA motor results in passage of each DNA strand though dedicated and different channels within the enzyme complex (9). A RecB-family nuclease domain lines the exit of each of these channels and stochastically cleaves the emerging single strands of DNA (9,10). Critical to the end resection reaction is the recognition of the recombination hotspot sequence Chi, and this occurs during translocation (11–13). Chi is recognized by the N-terminal region of the AddB subunit, which shares structural homology with UvrD-like SF1 helicases (9). The Chi recognition domain of AddB is arranged in series with the AddA motor, and ahead of the AddA nuclease domain. Therefore, it has been suggested that binding of Chi to AddB serves to sequester the 3′-terminated strand from engaging the AddA nuclease domain, thus attenuating nuclease activity specifically on this strand (9,14). It is thought that continued translocation and unwinding occurs downstream of Chi, resulting in processive degradation of the 5′-strand that leaves behind a recombinogenic 3′-ssDNA loop.

We have previously used a magnetic tweezers apparatus to investigate AddAB translocation on DNA. Under these conditions, in which there is a small restraining force on the movement of the enzyme, we showed that interaction of the translocating ssDNA with the Chi scanning domain is antagonistic to forward movement, giving rise to low-frequency pausing at Chi-like sequences (15). Furthermore, when AddAB encounters a locus containing 10 Chi sequences, it displays a non-exponentially distributed pause consistent with a multi-step mechanism for Chi recognition (15). These experiments also revealed a Chi-induced translocation rate change in the enzyme and showed that the pre- and post-Chi translocation rates were not correlated. Based on a limited number of single molecule observations, the net effect was to slightly decrease the average translocation rate of AddAB post-Chi. In this study, we have employed stopped-flow methods to further characterize the translocation behaviour of AddAB at and beyond Chi sequences. Using a combination of bulk triplex displacement and phosphate release assays, we have monitored DNA translocation and adenosine triphosphate (ATP) hydrolysis in real time, allowing us to understand how these activities are coupled before Chi and how this coupling is affected by encounter with recombination hotspot sequences.

## MATERIALS AND METHODS

### Protein expression and purification

Wild-type AddAB and AddAB^F210A^ were purified as described before (9). Phosphate binding protein was prepared according to the method of Brune et al. (16).

### DNA substrates

All DNA substrates were made by polymerase chain reaction (PCR) using one 5′-biotinylated primer (ATDbio) and one standard primer with Phusion DNA polymerase (NEB) following manufacturer's instructions. When this DNA is incubated with streptavidin, AddAB cannot initiate unwinding from the biotinylated end (8). PCR products were purified using a QIAquick PCR purification kit (Qiagen) or resolved and cut out from 1% TAE agarose gels at 4°C. DNA was extracted from gel slices using the Gene Jet gel extraction kit (Thermo) following manufacturer's instructions. Gel extracted DNA was dialysed overnight against a buffer containing 10 mM tris pH 8.0 and 0.1 mM EDTA to remove traces of GuHCl. DNA concentrations were calculated using an extinction coefficient of 6500 M^−1^ cm^−1^ nucleotide^−1^ at 260 nm.

Triplex displacement substrates were derived from the parent plasmids pSP73-JY0-TFO and pSP73-JY10-TFO (17). Details of the primer combinations used to make each substrate can be found in Supplementary Table S1. For experiments where the distance between Chi and the triplex binding site was varied, one deletion and two insertion derivatives of the pSP73-JY10-TFO plasmid were made which are referred to as pSP73-JY10-TFO del, pSP73-JY10-TFO i2 and pSP73-JY10-TFO i1. Briefly, a 1057 bp deletion was made in plasmid pSP73-JY10-TFO by PCR with outward facing phosphorylated primers. The two insertion mutant derivatives were made by amplifying the regions 87–1077 bp (i2) and 87–1878 bp (i1) from the *Escherichia coli uvrD* gene with primers that incorporate PstI restriction sites at each end. PCR products were cleaved with PstI (NEB) and ligated into PstI linearized pSP73-JY10-TFO. A single correctly orientated Chi sequence at position 143 bp in the *uvrD* insert was removed using the Quick change II XL site-directed mutagenesis kit (Stratagene) following the manufacturer's instructions. All pSP73-JY10-TFO derivatives were sequenced to ensure correct orientation of the insertions and the absence of unwanted mutations.

Phosphate release experiments were conducted on substrates derived from the parent plasmids pSP73-JY0-BbvCI-Superchi-Forward/Reverse and pSP73-JY10 (15,17). See Supplementary Table S1 for primer combinations used to make DNA substrates.

### Triplex displacement assays

DNA substrates (25 nM) containing a single triplex binding site were annealed overnight at 20°C to a 5′ tetramethylrhodamine (TAMRA) labelled triplex forming oligonucleotide (5′-TTC TTT TCT TTC TTC TTT CTT T, MWG, 100 nM) in a buffer containing MES (12.5 mM, pH 5.5) and MgCl_2_ (10 mM). Free triplex forming oligonucleotide (TFO) was removed by passage through a S400 spin column (GE healthcare) that was pre-equilibrated in triplex annealing buffer. Annealed substrates were kept on ice prior to dilution into reaction mixes. DNA substrates (2 nM) were incubated with streptavidin (100 nM, Sigma) in a buffer containing BSA (100 μg ml^−1^, Sigma), tris-acetate (25 mM, pH 7.5), magnesium acetate (2 mM) and DTT (1 mM). AddAB enzymes (10 nM) were incubated in this solution at 37°C for 2 min before mixing against an equal volume of a solution containing AddA^K36A^B (200 nM), ATP (1 mM), BSA (100 μg ml^−1^, Sigma), tris-acetate (25 mM, pH 7.5), magnesium acetate (2 mM) and DTT (1 mM). These two solutions were rapidly mixed using a stopped-flow device (SF-61 SX2, TGK scientific) and the resulting fluorescence was recorded. TAMRA was excited at 547 nm with the slits set at 5.4 nm and the fluorescence above 570 nm was recorded. Data were normalized using the maximum fluorescence end point.

Triplex data were fit to the following equation which defines triplex displacement *Y*, as being the sum of two offset exponential terms with *X*-axis offsets at times *T*1 and *T*2, with amplitudes *A*1 and *A*2 and apparent rate constants of triplex displacement *k*1 and *k*2:

(1) \begin{eqnarray*}
{Y} = (X > T1) \times [{A1( {1 - ( {e^{({ - k1(X - T1)})}})})}]\nonumber\\
 + (X > T2) \times [{A2({1 - ({e^{({ - k2(X - T2)})}})})}]
\end{eqnarray*}

Amplitudes from data obtained on Chi-0, 1, 2 and 3 substrates were fit to the following equation that describes the proportion of enzymes that recognize Chi, *Y*, as function of the probability of recognizing Chi, *P*, and the number of Chi sequences on a DNA substrate, *X*:

(2) \begin{equation*}
Y = [(100((1 - P)^X ))] - {\rm background}
\end{equation*}

A background value for the second phase amplitude was determined on Chi-free DNA and this was always less than 11% of the total fluorescence change. The standard error associated with fitted parameters is reported in Supplementary Tables S2–S4. We note that the standard errors of the lag times obtained from the fits are maximally 0.46% of the total lag time, whereas the standard errors associated with the linear fits that are used to determine the translocation rates are typically 1–4% of the determined translocation rates. Therefore, the majority of the error associated with determining translocation rates using this approach comes from linear regression of the lag time data for the different substrates used, not from the individual fits to the triplex displacement data.

### ATPase assays

ATPase measurements were performed in a stopped-flow apparatus (SF-61 SX2, TGK scientific) under similar conditions to triplex displacement experiments. DNA substrates (0.3 or 2 nM) were incubated with streptavidin (3 or 20 nM, respectively, Sigma) in a buffer containing BSA (100 μg ml^−1^ Sigma), tris-acetate (25 mM, pH 7.5), magnesium acetate (2 mM), DTT (1 mM) and a phosphate mop system consisting of 7-methylguanosine (200 μM, Sigma) and 0.01 units μl^−1^ bacterial PNPase (Sigma). This solution was left at room temperature for 30 min to allow efficient phosphate removal by PNPase before placing it on ice. The activity of the phosphate mop is not significantly active during the time scale of experiments described below (Supplementary Figure S7). AddAB enzymes (3 or 20 nM) and MDCC-PBP (5 μM) were added to DNA mixes and incubated at 37°C for 2 min before mixing against an equal volume of a solution containing heparin (1 mg ml^−1^, Sigma), ATP (1 mM) BSA (100 μg ml^−1^, Sigma), tris-acetate (25 mM, pH 7.5), magnesium acetate (2 mM), DTT (1 mM), 7-methylguanosine (200 mM, Sigma), 0.01 units μl^−1^ bacterial PNPase (Sigma) and MDCC-PBP (5 μM). This solution was also pre-incubated at room temperature for 30 min prior to MDCC-PBP addition. MDCC-PBP was excited at 436 nm with the slits set at 1.8 nm and resulting fluorescence above 455 nm was recorded. The change in fluorescence associated with phosphate production was calibrated by titrating known amounts of K_2_HPO_4_ against MDCC-PBP in reaction buffer including heparin but omitting PNPase and AddAB enzyme. The relationship between [phosphate] and the fluorescence signal was linear up to 2 μM phosphate and the maximum phosphate concentration measured in our experiments was 1.2 μM. Data describing the relationship between phosphate release amplitude and DNA length were fit to a linear equation or to the following equation that describes the amount of ATP hydrolyzed, *Y*, as a function of DNA length, *X*, with a finite processivity, *N* (the average number of base pairs unwound per AddAB binding event (18)), and coupling efficiency, *M:*

(3) \begin{equation*} Y = M\left[ {N - \left( {N\left( {\left( {\frac{{(N - 1)}}{N}} \right)^X } \right)} \right)} \right]
\end{equation*}

Breakpoints in ATPase data were determined objectively by fitting data around the ATPase rate transition to the following equation using GraphPad Prism:

(4) \begin{equation*}
\begin{array}
{*{20}c} {{Y}1 = ({\rm slope}1)*{X} + {C}}\\
{{\rm Yat}{\,X}0 = ({\rm slope}1)*{X}0 + {c}} \\
{{Y}2 = {{\rm Yat}{\,X}}0 + ({\rm slope}2)*({X} - {X}0)} \\
{{Y} = {\rm IF}(({X} < {X}0),{Y}1,{Y}2)} \\
\end{array}
\end{equation*}

The Chi-dependent decrease in ATPase rate was estimated using the following equation:

(5) \begin{equation*} Y = 100 \times \left[ {\left( {1 - \frac{a}{b}} \right) - \left( {1 - \frac{c}{d}} \right)} \right]
\end{equation*}

Equation (5) calculates the gradient of the data within a 200 ms timeframe immediately before and after the Chi-dependent decrease in ATPase activity. These gradients are referred to as ‘*b*’ and ‘*a*’, respectively. The same procedure is conducted on Chi-free DNA to give gradients ‘*d*’ and ‘*c*’. The quotient of these gradients gives the fold change in ATPase rate around the breakpoint. Equation (5) therefore reports the percentage decrease in ATPase activity after Chi has been recognized, which is corrected for the background (Chi-independent) decrease in ATPase activity that occurs over the 200 ms time window.

## RESULTS

### Characterizing AddAB translocation on Chi-free DNA

Translocation along DNA by AddAB was measured using triplex displacement (19). This assay places a 5′-TAMRA labelled TFO at a specific DNA locus such that, when translocating AddAB enzymes encounter the triplex, the TFO is displaced resulting in a fluorescence change. DNA substrates were blocked at one end via a biotin:streptavidin complex. This ensures that translocation proceeds unidirectionally from the non-biotinylated DNA end only and greatly simplifies the interpretation of the resulting kinetics (8). Experiments were performed under single turnover conditions by including the helicase mutant, AddA^K36A^B. This mutant traps free DNA ends to ensure that triplex displacement is caused predominantly by AddAB enzymes that were prebound to DNA before mixing in the stopped flow, and not by enzymes that reinitiated translocation by rebinding to free DNA ends, i.e. single turnover conditions (8). Triplex displacement curves are typically analysed by fitting data to an offset exponential (19). For reasons that will become apparent below (especially for the analysis of Chi-containing substrates), all triplex displacement data were analysed semi-quantitatively by fitting to the sum of two offset exponentials (Equation 1). The best-fitting parameters for all data that were fit to this equation can be found in Supplementary Tables S2–S4. This equation approximately describes triplex displacement occurring from two kinetically distinct populations of translocating enzymes.

**Figure 1.**
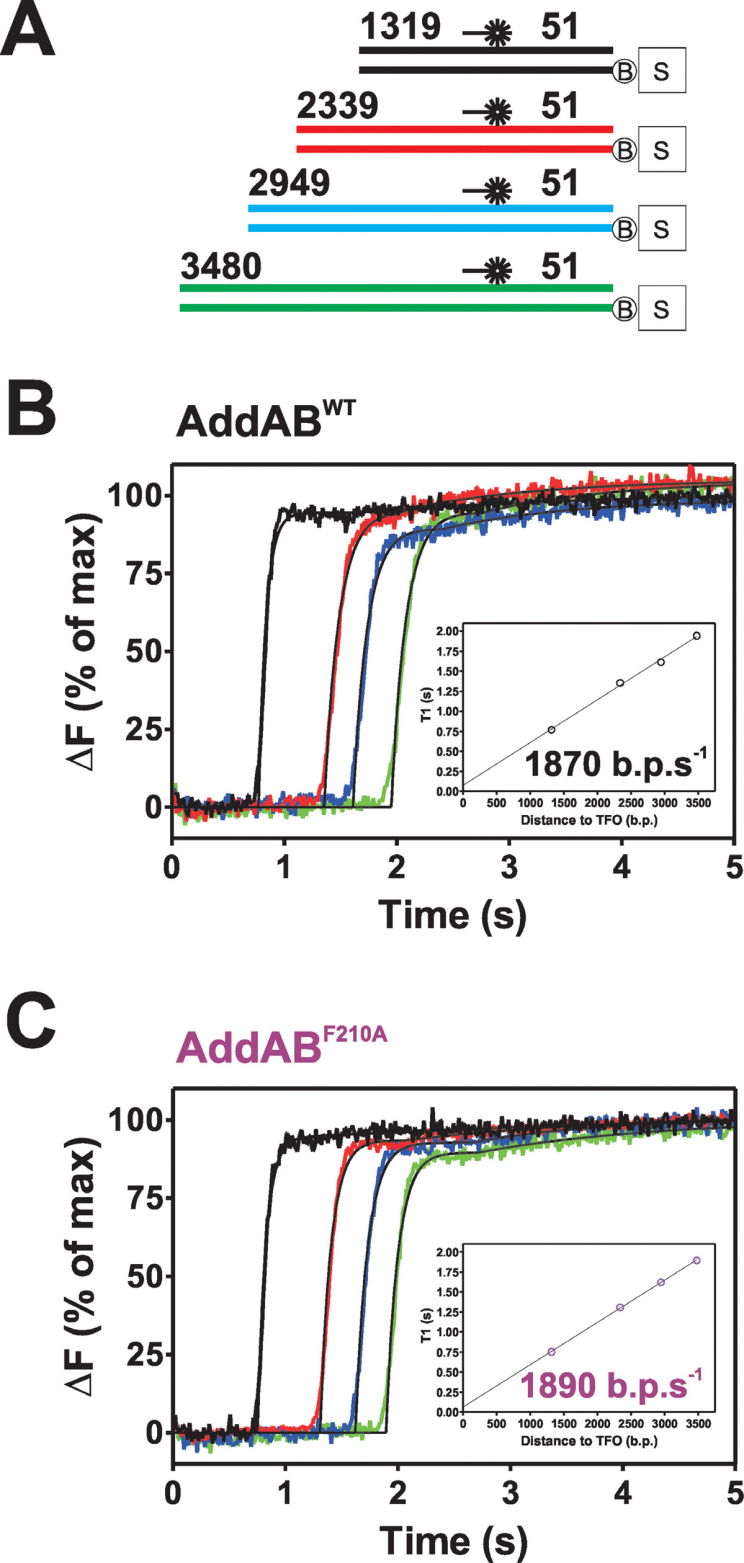
AddAB^F210A^ translocates normally on Chi-free DNA. (**A**) Schematic of substrates used for triplex displacement experiments; the colours used match the displacement curves shown in B and C. (**B**) Triplex displacement by wild-type (WT) AddAB. 2 nM DNA molecules were prebound by 10 nM AddAB enzymes. Reactions were initiated by mixing with an equal volume of ATP (1 mM) and AddA^K36A^B (200 nM) at 37°C. Data are the average of at least three transients and normalized to the endpoint of fluorescence. (**C**) Triplex displacement by AddAB^F210A^ using the same conditions as (A). Data are the average of at least three transients and normalized to the fluorescence endpoint. Insets show T1 lag times obtained from a fit to data in (A and C) using Equation (1). These values are plotted against the distance to the TFO and fit to a linear function (black lines), the gradient of which equates to the translocation rate.

Triplex displacement experiments were first performed on four Chi-free DNA substrates with a variable spacing between the free DNA end and triplex binding site (Figure 1A). Experiments were conducted both with wild-type AddAB and with a mutant protein AddAB^F210A^, which is severely defective in its response to Chi (20). Previous experiments have suggested that this mutant protein displays normal resection of Chi-free substrates, and so it would act as a useful control for any effects Chi might have on translocation (20). On Chi-free DNA, triplex displacement is preceded by a lag, the duration of which corresponds to the time taken for AddAB enzymes to initiate and translocate to the triplex (Figure 1B and C). A small second phase of displacement (∼10% of the total amplitude) is also apparent in both wild-type and mutant traces. This is likely to represent a small population of AddAB molecules that, initiate, translocate and/or interact with the triplex differently. The convolution of two offset exponentials was fit to the data, principally in order to extract the first phase lag time T1, but also to semi-quantitatively describe the time course of triplex displacement. T1 lag times were obtained for each of the four substrates and these values were plotted against the distance between the AddAB binding site and the triplex (Figure 1, insets). The linear relationship between lag time and distance travelled allows the determination of the translocation rates of the wild-type and mutant enzymes which were found to be 1870 ±70 bp s^−1^ and 1890 ±21 bp s^−1^, respectively (Figure 1B and C, insets). These values agree well with a previous published value of 1800 bp s^−1^ for the wild-type enzyme measured under similar conditions (17). Having established that the AddAB^F210A^ mutant translocates normally on Chi-free DNA, subsequent experiments were performed on substrates containing a ‘Chi locus’ engineered in-between the free DNA end and the triplex binding site, and incorporating either zero, one, two or three closely spaced Chi sequences.

### Chi sequence recognition delays triplex displacement

**Figure 2.**
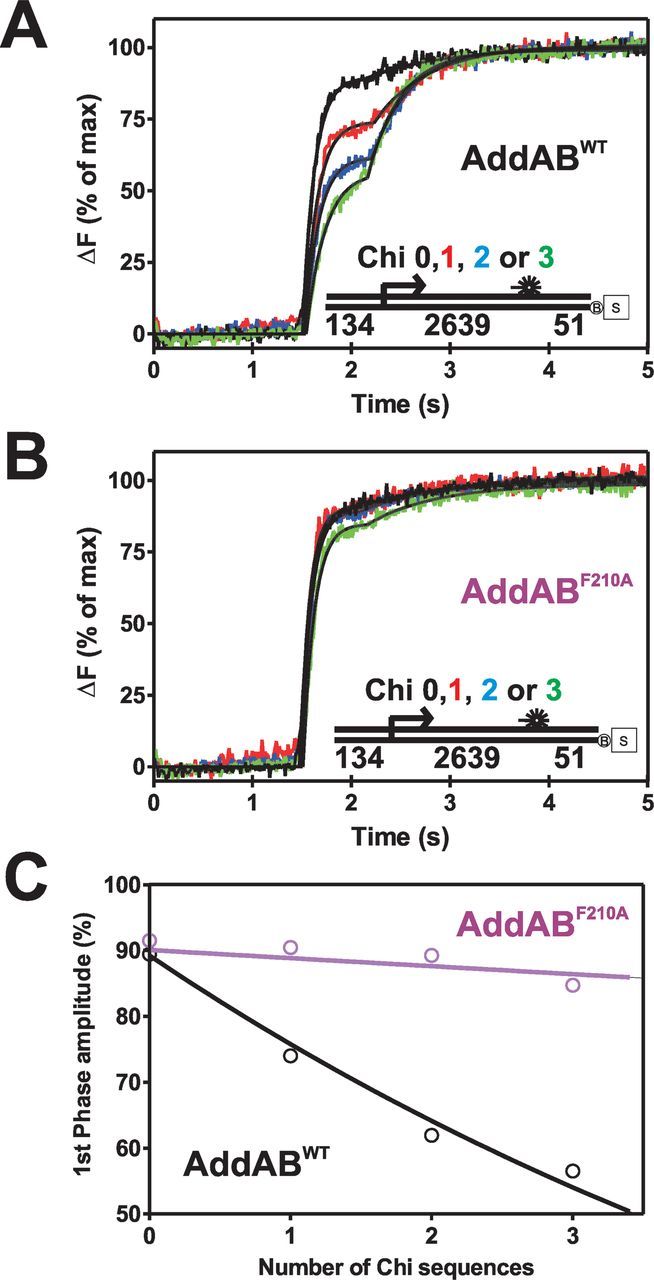
The recognition of Chi results in a net decrease in the translocation rate of AddAB. (**A**) Triplex displacement by wild-type (WT) AddAB on Chi-containing DNA, the number of Chi sequences in the Chi locus (right-facing arrow) and its location are indicated on the substrate schematic shown as an inset. DNA molecules (2 nM) blocked on one end by a biotin:streptavidin complex were prebound by 10 nM AddAB enzymes for 2 min at 37°C before mixing against an equal volume of ATP (1 mM) and AddA^K36A^B (200 nM). Data are the average of at least three transients and normalized to the fluorescence endpoint. (**B**) Triplex displacement by AddAB^F210A^ on Chi-containing DNA under the same conditions as (A). Data are the average of at least three transients and normalized to the endpoint of fluorescence. (**C**) Plot of first phase amplitude as a function of the number of Chi sequences yields the apparent probability of recognizing Chi by fitting to Equation (2).

Experiments with wild-type AddAB and Chi-containing DNA substrates show markedly different TFO release kinetics, in that the TFO is now released in two well-resolved phases, the first of which occurs at the same time as on Chi-free DNA (Figure 2A). This is indicative of two separate, tightly distributed, populations of AddAB arriving at the triplex at different moments in time. Recognition of Chi is an inefficient process (10,14,17,21–24), and so the response to Chi is expected to be dose dependent and this is clearly the case. As the number of Chi sequences at the Chi locus is increased, the relative amplitude of the first phase of triplex release decreases and that of the second phase increases (Figure 2A and C). This strongly suggests that the second phase of triplex release is caused by translocation of enzymes that have successfully recognized Chi sequences. In further support of this idea, equivalent experiments performed with the Chi recognition mutant AddAB^F210A^ show that the second phase of triplex displacement is always small such that it is comparable to that seen on Chi-free DNA. This observation also suggests that the second phase of triplex displacement observed on Chi-free DNA with wild-type AddAB does not originate from a sub-population of enzymes that recognize sequences other than the *bona fide* Chi sequence. The relationship between the triplex displacement amplitudes and the number of Chi sequences was exploited in order to obtain an apparent probability of Chi recognition. The first phase amplitude was plotted as a function of the number of Chi sequences and fit to Equation (2) (Figure 2C), which describes the process of Chi recognition as being a stochastic event with no cooperativity between successive Chi recognition attempts. The data are well described by this fit, yielding a probability of 0.13 ±0.02 and 0.01 ±0.004 for the recognition of Chi by the wild-type and AddAB^F210A^ enzymes, respectively. We conclude that those enzymes that have recognized Chi, which we shall refer to as AddAB*, take longer (0.67 s on average for these substrates) to displace the triplex, which is consistent with the net translocation rate being faster on Chi-free DNA. Interestingly, although all of the Chi-containing substrates yield similar values for the T1 and T2 lag times, it is clear that there is a trend for the value of T1 to increase slightly as a function of the number of Chi sequences (Supplementary Table S3). This raises the possibility that the presence of Chi delays triplex displacement by enzymes that nevertheless did not apparently recognize Chi. We will return to this point in the discussion.

### Modelling the effect of Chi on DNA translocation

**Figure 3.**
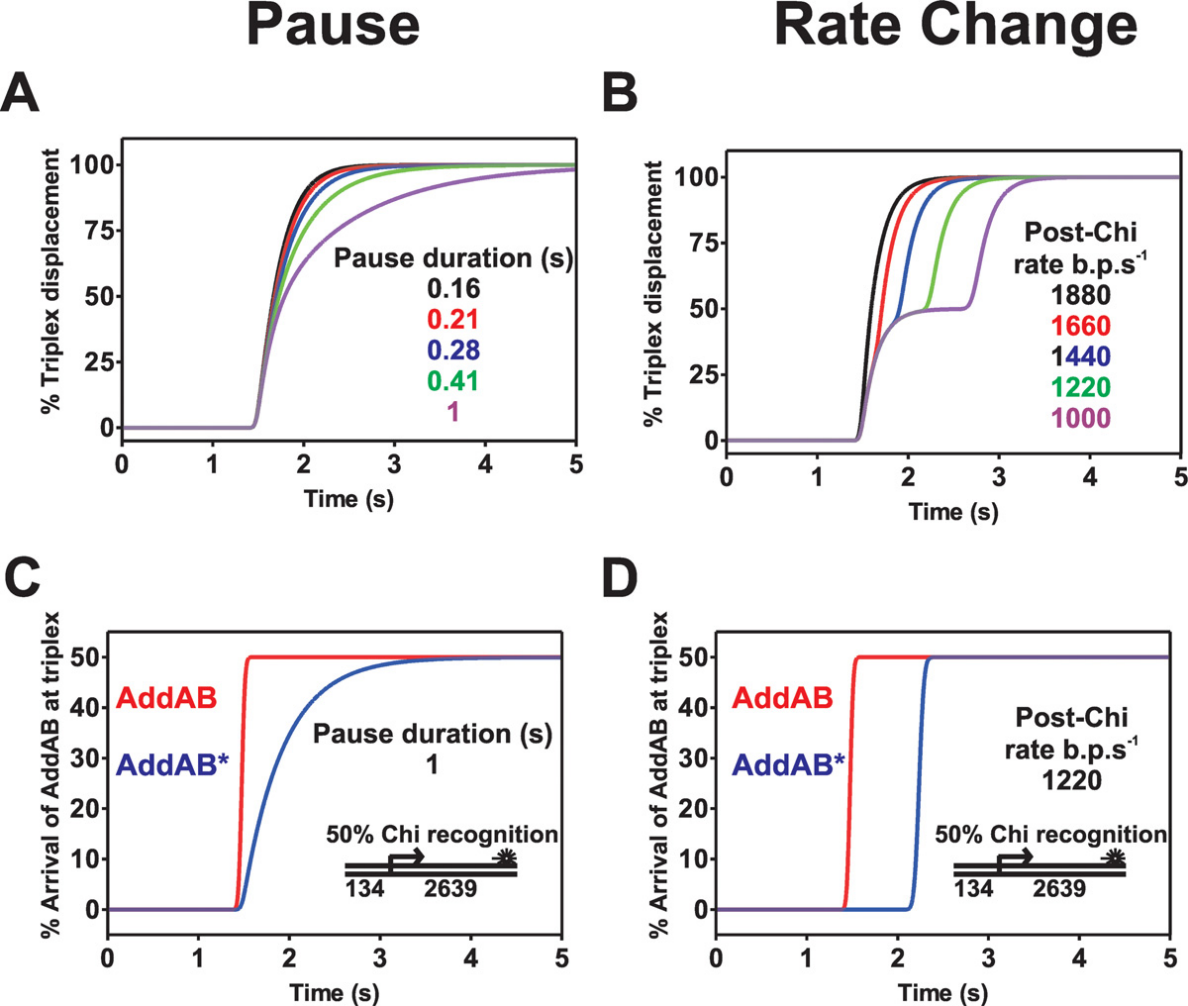
The single pause at Chi model does not accurately describe experimental triplex displacement profiles. (**A**) Simulation of triplex displacement when AddAB pauses at Chi. AddAB translocates at 1880 bp s^−1^ and the pause duration at Chi is increased from 0.16 s (black) to 1 s (purple). (**B**) Simulations of the rate change model varying the post-Chi translocation rate from 1880 bp s^−1^ (black) to 1000 bp s^−1^ (purple). The triplex is displaced with a first-order rate constant of 6 s^−1^ in both models. All models assume 50% Chi recognition at the Chi locus. (**C**) and (**D**) The arrival of enzymes that did not recognize Chi (AddAB, red line) and enzymes that did recognize Chi (AddAB*, blue line) at the triplex. For further details see Supplementary Discussion 1. A schematic of the *in silico* substrate used for modelling in this figure is shown in both panels (C) and (D).

On the basis of the data presented above alone, there are several possible explanations for the delay in triplex displacement caused by recognition of Chi sequences. The AddAB complex might stall transiently at or beyond Chi, the translocation rate of the complex might be decreased beyond Chi, or there might be a mixture of both effects as has been shown by single molecule experiments under different conditions (15). It is also formally possible that the delay arises from an artefactual effect, such as the Chi-modified form of the enzyme displacing the triplex forming oligonucleotide via a different mechanism. Modelling the triplex displacement kinetics can help to distinguish between these scenarios and inform the design of experiments that more clearly differentiate between them. Initially, two extreme scenarios were simulated, which are both variations on classical ‘n step sequential’ models that describe the translocation of AddAB along a 1D DNA lattice as a series of irreversible first-order steps (25). In one model, a proportion of the AddAB population (50%; approximately mimicking recognition of three tandem Chi sequences) undergo a single stochastic pause at Chi and in the other model, the same proportion do not pause at Chi, but slow down after Chi recognition (i.e. each step along the DNA takes longer to accomplish beyond the Chi sequence). Simulations of these two models are shown in Figure 3 and further details about their design and implementation can be found in Supplementary Discussion 1.

There is a very striking difference between the triplex displacement profiles of the two models. In the pause model triplex displacement is biphasic, but the second phase is continuous with the first (Figure 3A). This is because the fastest molecules in both populations of AddAB arrive at the triplex simultaneously, but the enzymes that have recognized Chi (AddAB*) have a wider and retarded distribution along the DNA (Figure 3C). This results in a slower apparent triplex displacement rate for the second phase. In stark contrast, the rate change model yields two temporally distinct phases of triplex displacement that more closely resemble the experimental data. This can again be understood by comparing the distributions of the individual populations at the triplex. In the rate change model, the AddAB* enzymes remain tightly grouped, but are displaced to longer timepoints (Figure 3D). This means that the triplex displacement phase caused by enzymes that have not recognized Chi is largely complete before the triplex starts getting displaced by AddAB* enzymes. A comparison of these two models with the experimentally observed triplex displacement profiles shows that a single stochastic pause at Chi is an inadequate description of the experimental data, and this model can be excluded.

An alternative pause model that could result in the delay of observed triplex displacement would involve a non-exponentially distributed pause at Chi. Such a phenomenon could occur if multiple kinetic processes (with similar time constants) occurred during the pause and this gives rise to triplex displacement profiles that look similar to the rate change model (data not shown). However, as will be demonstrated below, it is possible to distinguish between all of these models if the distance between the Chi locus and the triplex is varied. This is because, in rate change models, the delay caused by Chi recognition is proportional to the distance between Chi and the triplex, whereas in any pause model the delay is independent of this distance. Further discussion, including simulations relevant to this point, and the efficacy of semi-quantitative analysis methods to extract the delay values, can be found in the Supplementary Discussions 2 and 3.

### The translocation rate of AddAB decreases following Chi recognition

**Figure 4.**
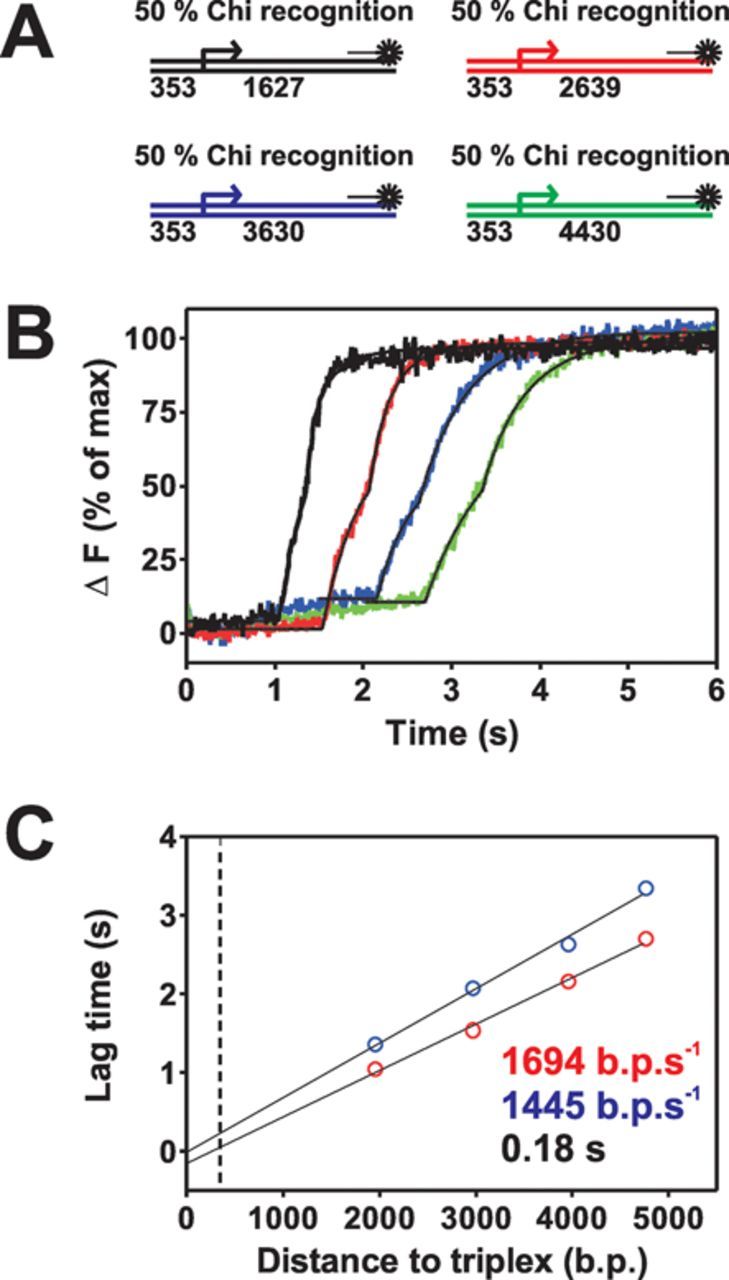
The translocation rate of AddAB decreases following Chi recognition. (**A**) Schematic of substrates used in this experiment. (**B**) Triplex displacement with wild-type AddAB on Chi-containing DNA with variable distances between Chi and the triplex binding site. The distances and colour coding are identical to those in substrates used for modelling (Supplementary Figure S3). DNA molecules (2 nM) blocked on one end by a biotin:streptavidin complex were prebound by AddAB enzymes (10 nM) for 2 min at 37°C before mixing against an equal volume of ATP (1 mM) and AddA^K36A^B (200 nM). Data are the average of at least three transients and normalized to the fluorescence endpoint. Black lines indicate fits to the data using Equation (1). The blue and green traces are only fit from 1.5 and 2 s onwards, respectively (see the main text for discussion). (**C**) Plot of the the first and second phase lag times, T1 (red) and T2 (blue) as a function of DNA length. Linear fits to these data yield values for the pre- and post-Chi translocation rate. The black dotted line is the position of the Chi locus.

Based on the modelling described above and to distinguish between our different models, we next investigated the triplex displacement kinetics associated with the family of substrates shown in Figure 4A. Three closely spaced Chi sequences were present in all of the substrates to give a substantial second phase of triplex displacement (∼50%), but the distance between the Chi sequences and the triplex was varied from ∼1600 bp to ∼4500 bp. As expected, on all substrates the triplex displacement is essentially biphasic (Figure 4B). On the two longest substrates (blue and green lines), a small amount of triplex displacement (∼12%) occurs at an unexpected time (∼0.4 s). These two substrates contain the same large DNA insertion and so this small signal possibly reflects a weak secondary triplex binding site which was not identified from sequence analysis. For each substrate, the data were fit to the sum of two offset exponentials to derive lag times (T1 and T2) for the two triplex displacement phases. These values were then plotted against the distance to the triplex and both sets of data were well fit using linear regression. The divergence of the lines that are fit to the values of T1 and T2 is consistent with a rate change model (Figure 4C, Supplementary Figures S2 and S6B) and the gradients of these lines yield values for the translocation rates of 1694 ± 83 and 1445 ± 80 bp s^−1^ for AddAB and AddAB*, respectively. We conclude that the Chi-modified enzyme, AddAB*, translocates ∼15% more slowly than AddAB and that this is mainly responsible for the delay in triplex observed in our experiments. However, these data also provide weaker evidence for a pause at Chi, because the fitted line for the T2 lag times does not intercept with the fit to the T1 times at the position of the Chi locus (353 bp; dotted line in Figure 4C and Supplementary Figure S6B). This value is small with the offset of the fitted lines at Chi suggesting a pause of 180 ± 3.5 ms. Unlike the values for T1 and T2 lag times (and therefore the AddAB and AddAB* translocation rates) which are highly reproducible in independent experiments (see Supplementary Figure S6 for a second data set), the value for the pause is subject to significant error due to extrapolation, and a second data set gave a value of 65 ±1.2 ms. Taken together, the data suggest that Chi recognition reduces the translocation rate of AddAB beyond Chi and causes a brief pause (presumably at Chi) on the order of 100 ms.

### The rate, but not the extent, of ATP hydrolysis is decreased following the recognition of Chi

To determine if the Chi-dependent translocation rate decrease also manifests itself as a decrease in ATP hydrolysis by the AddA motor, or whether translocation may become more loosely (or indeed tightly) coupled to ATPase activity after Chi, we measured ATPase activity associated with directional translocation and unwinding of DNA. This was achieved using a biosensor for inorganic phosphate, MDCC-PBP (26). First, conditions were found where ATPase activity that was associated with directional translocation could be observed. This was achieved by performing single turnover ATPase stopped-flow experiments in which AddAB was prebound to DNA and rapidly mixed against ATP and heparin. Heparin competes with dsDNA for binding to AddAB, but does not stimulate ATP hydrolysis (Supplementary Figure S7), and so largely constrains the measurement to ATP hydrolysis associated with translocation of AddAB enzymes that were bound to DNA ends at time zero.

**Figure 5.**
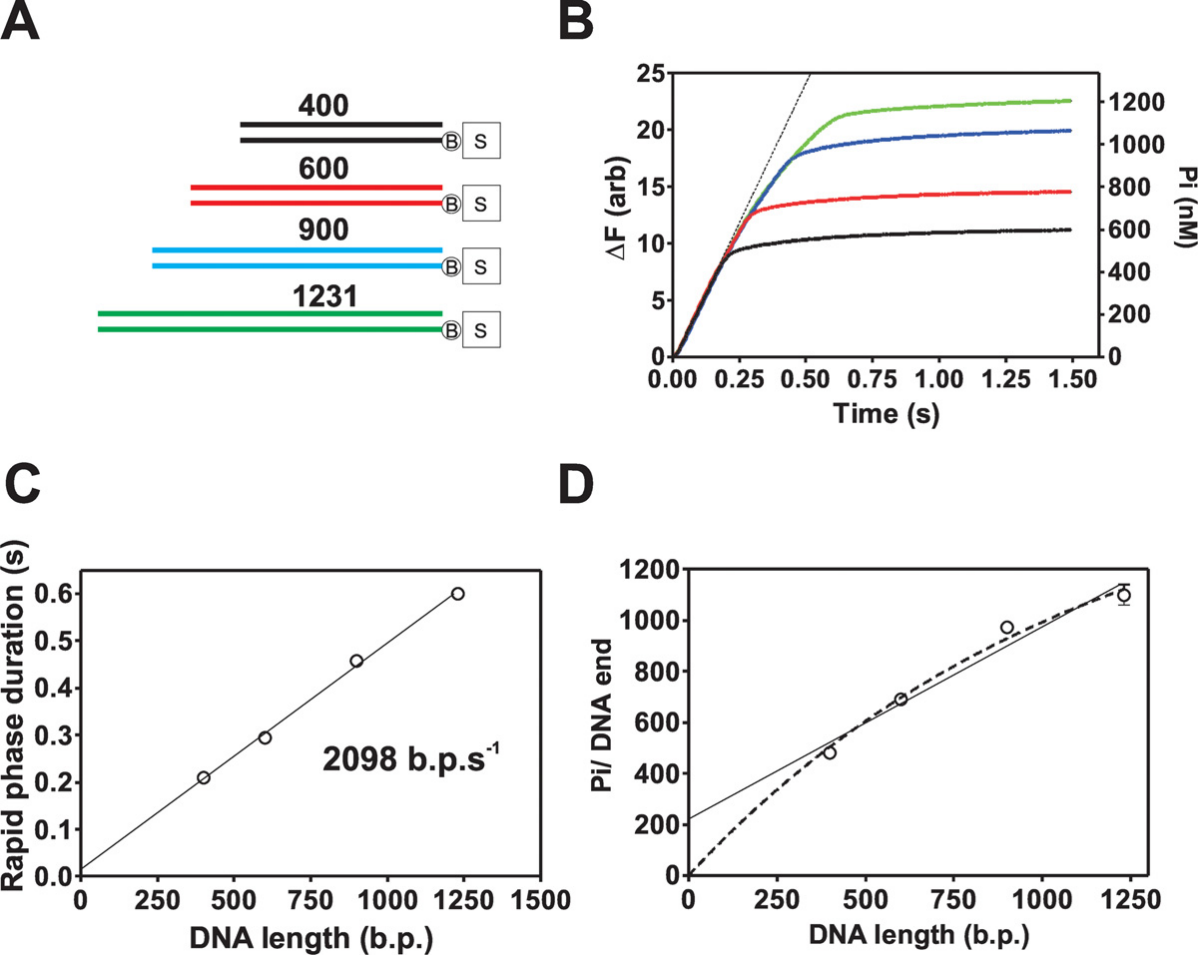
Real-time observation of the ATPase activity that powers coupled translocation and unwinding of DNA. (**A**) Schematic of biotin:streptavidin blocked substrates used in these experiments. (**B**) Transients of ATPase measurements performed on DNA of differing lengths. DNA substrates (2 nM) were prebound by AddAB enzymes (20 nM) before mixing against an equal volume of ATP (1 mM) and heparin (1 mg ml^−1^). Data are the average of at least three traces. The black dotted line is a simulation of a straight line based on an average initial ATPase rate of 2629 ATP s^−1^. (**C**) The duration of the rapid phase as a function of DNA length is linear indicating that this phase is associated with DNA translocation. Data are the average of three independent experiments, and the error bars are too small to be seen by eye. (**D**) The number of phosphate molecules produced per AddAB binding site as a function of DNA length provides information on the coupling efficiency of AddAB when it translocates along DNA. Two fits were performed to the data, the solid black line is a linear fit to the data and the dashed line is a fit using Equation (3). Data are the average of three independent experiments. Error bars represent the SEM.

ATPase activity was initially characterized on Chi-free DNA using DNA substrates of variable length (Figure 5A and B). ATPase activity proceeds with a rapid phase which is succeeded by a much slower phase which is heparin-sensitive (Supplementary Figure S7). As expected, the duration and amplitude of the rapid phase increase with DNA length which is characteristic of a directionally translocating motor protein. The rapid phase also displays some downwards curvature (see deviation from the dotted line in Figure 5B), indicating that the ATPase rate is not constant over the entire time course, and progressively decreases as the enzyme population progresses along the substrate. However, the initial ATPase rates are very similar across all substrates, giving an average value of 2629 ± 240 ATP s^−1^ AddAB^−1^. The duration of the rapid phase was calculated objectively by fitting the data around the apparent breakpoint in the data. The rapid phase duration is proportional to the DNA length yielding a translocation of 2098 ± 69 bp s^−1^. This is in reasonable agreement with the translocation rate obtained from triplex displacement experiments (Figures 2 and 5C), and further demonstrates that the rapid phase is associated with ATPase activity that is coupled to directional translocation along DNA. A simple method to determine the apparent efficiency or coupling ratio, of the AddA motor is to simply take the quotient of the initial rapid phase ATPase rate (2629 ± 240 ATP s^−1^ AddAB^−1^) and translocation rate (2098 ± 69 bp s^−1^ AddAB^−1^), which yields a coupling efficiency of 1.25 ± 0.12 ATP bp^−1^. However, this rate could also include ATPase activity that is not associated with translocation and therefore report an inflated coupling efficiency. Therefore, the amplitude of the rapid phase was plotted against the length of the DNA substrate (Figure 5D) to allow the coupling efficiency to be determined without this prior assumption. The simplest analysis is to fit a linear equation to the data shown in Figure 5D which assumes that the processivity of AddAB is infinite, and this returns a coupling efficiency of 0.75 ± 0.11 ATP bp^−1^. This fit has a large *Y*-axis intercept which could indicate that ∼200 ATP molecules are hydrolyzed per DNA molecule in a process that does not result in movement along DNA (e.g. initiation of translocation or futile ATPase activity at the distal DNA end). However, because the linear fit to the rapid phase duration intercepts the *Y*-axis at 17 ms (Figure 5C), there is not realistically enough time for ∼200 ATP molecules to be hydrolyzed in a process that is not associated with DNA translocation. A different fit (Figure 5D, dotted lines) was performed using an equation that requires the fitted line to go through the origin (which assumes all hydrolysis is coupled to translocation) and also includes a term for finite processivity. This fit describes that data well, yields an apparent coupling efficiency of 1.4 ± 0.075 ATP bp^−1^, in agreement with the initial rate-based analysis and suggests that the processivity of AddAB is rather low under these experimental conditions (∼1.5 kb). It has been estimated that AddAB has a processivity of between ∼14 and ∼25 kb and a homologue from *Bacteroides fragilis* has a processivity of 14 kb (8,27). However the measurement of *B. subtilis* AddAB was in the presence of Chi sequences (the *B. fragilis* Chi sequence is unknown) which could stimulate the processivity of AddAB (8). We conclude that the coupling efficiency is between 0.75 and 1.4 ATP bp^−1^ which is broadly similar to the value of 1 ATP bp^−1^ that is expected based on structural and biochemical analysis of related SF1A helicase motors (28–33).

**Figure 6.**
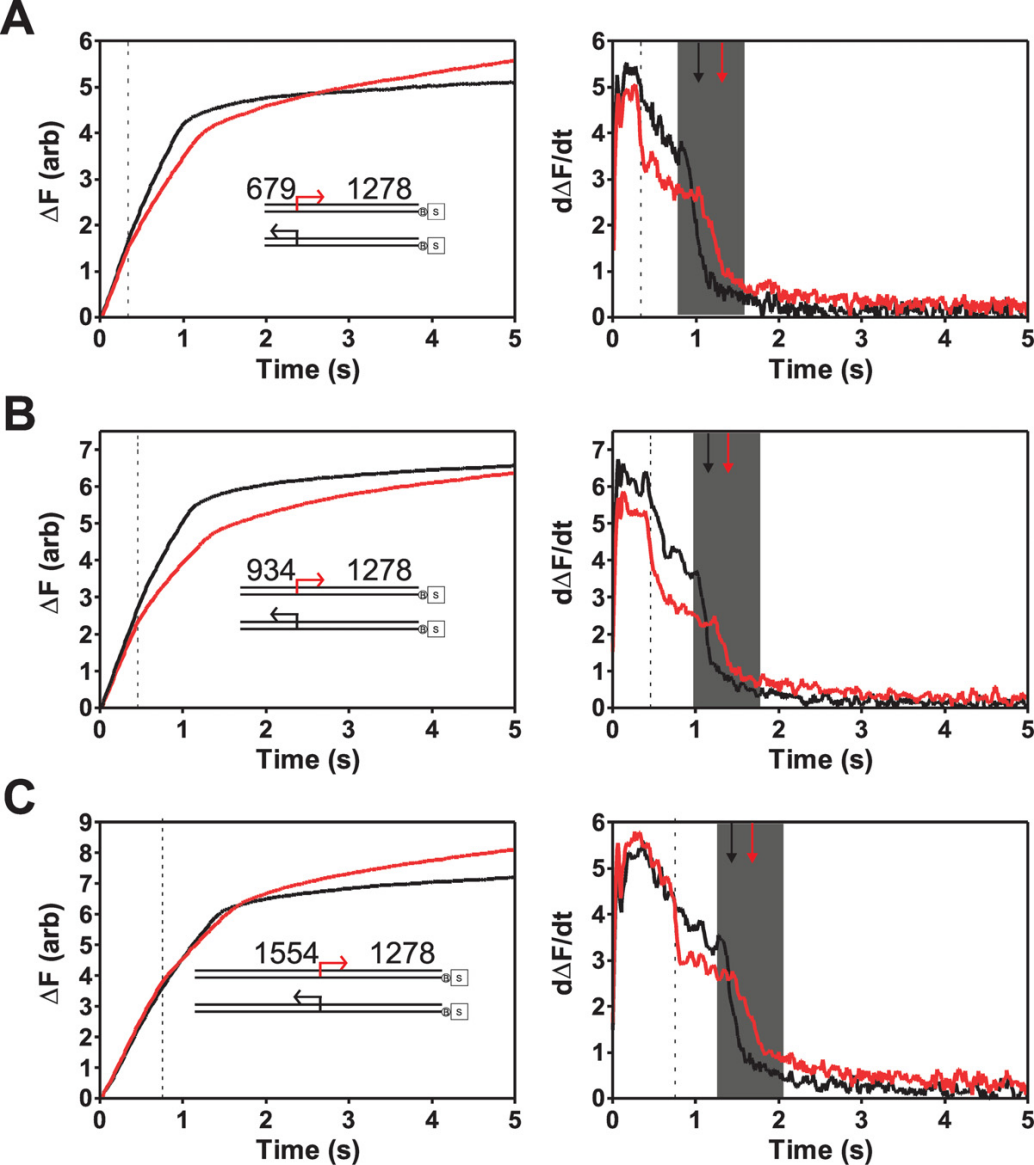
Chi recognition results in a sustained decrease in ATPase rate. (**A**)–(**C**) Left panels: single turnover phosphate release experiments performed on DNA substrates (inset) with a 10X Chi locus in either the correct (red) or incorrect orientation (black) for recognition. The distance between the free DNA end and Chi is indicated, and is different in each of the three pairs of substrates. DNA substrates (0.2 nM) were pre-incubated with AddAB enzymes (2 nM) for 2 min at 37°C before mixing against an equal volume of ATP (1 mM) and heparin (1 mg ml^−1^). Right panels: first derivative (i.e. the ATPase rate) of the data shown in the left panel. The data have been smoothed by averaging 13 neighbouring points. The grey shaded box indicates the region over which rapid ATPase activity terminates. The black and red arrows indicate the midpoint of these transtions for ‘reverse’ and ‘forward’ substrates, respectively. The black dotted lines show the predicted time (based on data in Figure 5C) for the arrival of AddAB at the Chi locus.

We next examined the effect of Chi recognition on ATP hydrolysis. Experiments were performed on a series of DNA substrates containing a short DNA locus (∼100 bp) that contains 10 Chi sequences either in the correct (‘forward’) or incorrect (‘reverse’) orientation for recognition by AddAB (15). This approach was used to maximize the level of Chi recognition and hence the proportion of AddAB* activity that we measure. This is particularly important because, unlike in the triplex displacement assay, ATPase measurements are continuous: they do not separate the behaviour of AddAB from AddAB* and instead we will observe the sum of the ATPase activities of the two populations. As expected, the ‘reverse’ substrates behaved qualitatively as Chi-free DNA. ATPase activity proceeds with a rapid phase that is followed by a very slow phase of ATP hydrolysis (Figure 6, black lines), and the breakpoint between these two phases corresponds to the time at which AddAB would reach the end of the substrate. Traces for the ‘forward’ substrates are distinct from those of ‘reverse’ substrates in that ATPase activity still occurs with a rapid first translocation phase, but this is *itself* biphasic, with the phosphate release abruptly slowing down during movement along the DNA. After the translocation phase, there is then a slower and prolonged second phase, but this is consistently faster than on ‘reverse’ substrates. The first derivatives of the ATPase traces clearly reveal the sudden change in ATPase rate before AddAB reaches the end of the DNA that occurs on the ‘forward’ substrates only. This rate decrease occurs precisely when AddAB would be predicted to arrive at the Chi locus based on the measured translocation rate (Figure 6, dotted lines and Supplementary Figure S8). This abrupt decrease in ATPase activity occurs against a background of a more gradual ATPase decrease during translocation that is present on traces for both ‘forward’ and ‘reverse’ substrates. This is consistent with a limited processivity of AddAB as was also suggested by the data shown above (Figure 5D).

A decreased ATPase rate beyond Chi is sustained until the ATPase rate drops dramatically again. This second transition, which presumably signals the arrival of the enzyme at the DNA end, is delayed relative to the ‘reverse’ substrates (compare black and red arrows in the shaded box in Figure 6). This is consistent with slower translocation beyond Chi as revealed by triplex displacement experiments. The overall picture is that beyond Chi, both the ATPase activity and the translocation rate of the AddAB enzyme are reduced. In order to compare the magnitude of these effects, we estimated the percentage reduction in ATPase activity after Chi by comparing the ATPase activity of AddAB for a 200 ms timeframe immediately before and after the Chi-dependent decrease using Equation (5). This yielded a value of 12.9 ± 4.3% for the rate decrease which is, within error, the same as the change in translocation rate (15.2 ± 0.76%) measured using triplex displacement. It should be noted that the decrease in ATPase rate after Chi is probably an underestimate of the true value because less than 100% of the AddAB population will have recognized Chi. The total amplitudes of ATP hydrolysis from a single round of translocation are not substantially different in any of the pairs of ‘forward’ and ‘reverse’ substrates (Supplementary Figure S9), which indicates that, although the recognition of Chi slows ATP hydrolysis, it does not result in a large change in the coupling between ATP hydrolysis and DNA translocation.

An unexpected additional observation from these data is that the post-translocation slow phosphate release phases on ‘forward’ substrates are reproducibly faster than their counterparts on ‘reverse’ substrates. It is known that AddAB–Chi complexes persist for far longer than the time it takes to translocate to the distal ends of the substrates used in these *in vitro* studies (21). Therefore, this phenomenon might reflect a desensitisation of the enzyme and its ATPase activity to the trapping effects of heparin, as it remains engaged with the reaction products post-translocation.

## DISCUSSION

The recognition of recombination hotspots dramatically transforms the biochemical properties of helicase–nuclease enzymes. Not only does Chi regulate the nuclease activity of these complexes but also the translocation and unwinding activities. The AddAB complex shows stimulation of helicase activity (i.e. DNA strand separation) by Chi. Previous studies established that this was largely due to an increase in the coupling of DNA translocation and unwinding (17), and were inconsistent with large changes to the translocation rate of AddAB. However, a recently published study characterizing AddAB movement on DNA using magnetic tweezers was sufficiently sensitive to detect pausing and rate changes induced by Chi (15). In that study, AddAB was found to pause at a site containing 10 Chi sequences for ∼1.5 s, and also to pause briefly at Chi-like sequences. Experiments with the AddAB^F210A^ mutant showed that both pauses were caused by the interaction of ssDNA sequences with the Chi recognition locus of AddB. The pause at Chi was non-exponentially distributed and was interpreted as the sum of the time constants required for conformation changes at Chi, or for the same events summed together with failed recognition attempts at the same Chi locus. Additionally, AddAB enzymes that passed over a correctly oriented Chi locus were found to change rate and on average decrease rate by ∼16%. This observation was made using correlation analysis of measured translocation rates before and after Chi as it was impossible to tell directly if a particular AddAB enzyme successfully recognized Chi (15). We stress that it is entirely possible for Chi recognition to cause both an increase in the net helicase (strand separation) activity (17) and a concomitant decrease in translocation rate, if the coupling between translocation and unwinding is increased to a greater extent than the translocation rate is decreased.

In this work, we have used bulk stopped-flow methods to monitor ATP hydrolysis and DNA translocation, and the effect of Chi on these processes, in real time. Using triplex displacement assays, we were able to compare the behaviour of enzymes that had not (AddAB) or had (AddAB*) recognized Chi on the same substrate, because these different enzyme populations were separated into kinetically distinct populations. These experiments showed that AddAB decreases translocation rate beyond Chi from ∼1700 bp s^−1^ to ∼1450 bp s^−1^ (a 15% rate decrease). It may be that this decreased translocation rate is the result of *in cis* interactions with the ssDNA loop that is thought to form following Chi recognition. However, the change in rate persists in the presence of P1 nuclease which would cut this loop structure (data not shown) (17). The data also provide evidence for a short pause at Chi, placing an upper limit of a few hundred milliseconds on the pause duration, but the form of the distribution of the pause duration cannot be determined from these data. An intriguing observation from the data presented here is the presence of a Chi-dependent increase in the values of the T1 lag times (Supplementary Table S3). We have argued that the T1 lag time is generated by enzymes that have not recognized Chi. In that context, this observation implies that enzymes which *do not* ultimately recognize Chi sequences are still slightly delayed in their progression along DNA by the presence of Chi sequences. This slight delay presumably reflects the kinetics of failed Chi recognition events and so one might also expect that the Chi-dependent pause duration would be dependent on the number of Chi sequences. However, the lack of correlation between the T2 lag times and the number of Chi sequences in our substrates (Supplementary Table S3) suggests that the pause at Chi associated with successful recognition is approximately constant regardless of the number of Chi sequences.

The pause at Chi measured here is much shorter than the 1.5 s delay reported in single molecule experiments (15). Those experiments were performed with substrates that contain 10 Chi sequences at the Chi locus, at a significantly lower temperature (20°C), and also with a restraining force of 3 pN, any or all of which might contribute to the longer pause. Indeed, magnetic tweezers experiments performed at 37°C display shorter pauses and a reduced pause frequency at Chi, probably because many pauses become too quick to measure (<0.3 s; C. Carrasco and F. Moreno-Herrero, personal communication). This indicates that the pause at Chi is temperature dependent as might be expected, although the reduction in rate seems to be apparent (and of a similar magnitude) at a range of different temperatures. We cannot exclude the possibility that the number of Chi sequences and/or the restraining force also influence the difference in the measured pause duration between the two experiments. This would require further investigation at the single molecule level because of the error associated with determining the pause duration at Chi accurately in bulk.

Real-time ATPase experiments showed that the AddA motor protein consumes approximately one ATP per base pair travelled. This is the value expected based on mechanisms for ssDNA translocation developed on the basis of crystal structures of SF1A helicases in the presence of different nucleotide analogues (28,29). Therefore, it is likely that ATP hydrolysis and DNA translocation are tightly coupled in AddAB. It was possible that the reduced translocation rate observed beyond Chi was the result of an uncoupling of translocation from the ATPase activity. However, we found that the ATPase activity was also downregulated following Chi recognition. The extent of this rate decrease was estimated at 13%, which approximately matches the observed change in translocation rate. Thus, following Chi recognition the coupling of ATPase to translocation seems to remain tight, despite the fact that the enzyme is thought to display an altered translocation mode in which the nascent ssDNA leaves the enzyme via a different pathway.

Two models can explain why AddAB decreases translocation rate after recognizing Chi while forward movement remains tightly coupled to ATP hydrolysis. One simple possibility is that Chi recognition induces a conformational change which directly affects the ATPase active site of AddA. When studied as isolated components, SF1A helicases display a very broad range of ATPase and translocation rates, even though the core motor unit is extremely similar between each family member. For example the helicases RecB, PcrA, UvrD, Rep and RepΔ2B translocate along ssDNA at rates of 803, 80, 189, 298 and 530 ntd s^−1^ (under similar solution conditions) (33–36). Such diversity suggests that the structural context of the motor could be important for controlling the rate at which the enzyme can translocate. Consistent with this idea is the observation that when RecB is in complex with RecC, its translocation rate increases by ∼20% (36). It therefore seems conceivable that a single-motor helicase could adopt multiple translocation modes via conformational switching. Indeed, the AddAB translocation rate displays both static and dynamic disorder at the single molecule level (15), and recent experiments with the *E. coli* RecBCD helicase–nuclease showed that this enzyme could adopt multiple translocation rates by sampling different conformational substates (37). An alternate model could be that AddAB becomes more prone to brief but frequent pausing after recognizing a Chi sequence, for example because of the inhibitory effects of pumping out the proposed ssDNA loop from within the complex. In that scenario, the pausing must also decrease the ATP hydrolysis rate, and this could occur if ATP hydrolysis was tightly coupled to the conformational changes that drive movement along ssDNA. A single molecule apparatus with extremely high temporal and spatial resolution might help resolve these mechanisms.

The functionally analogous RecBCD enzyme from *E. coli* also exhibits complex translocation behaviour when it encounters its cognate Chi sequence. The dual motor RecBCD enzyme switches lead motor when Chi is recognized, from the faster motor in the RecD subunit to that of the slower motor in the RecB subunit (38,39). It appears that AddAB may well have adopted the same translocation strategy as RecBCD, i.e. fast before Chi and slower after Chi, but the underlying basis for this is different between the two enzymes. This difference in translocation rate between AddAB and AddAB* provides a new and sensitive diagnostic tool with which to measure the response of AddAB to recombination hotpots. The results presented in this study also provide the first example, to the best of our knowledge, of ATPase activity modulation of a DNA motor as a result of specific ssDNA sequence recognition. By placing a DNA sequence recognition domain behind the translocase, the AddAB complex is able to modulate its motor activity in a sequence-specific manner. This principle might offer a general mechanism to control movement along DNA in either a sequence- or conformation-specific manner.

## FUNDING

ERC [206117 SM-DNA-REPAIR to M.D.]; Wellcome Trust New Investigator Award [100401/Z/12/Z to M.D.]; BBSRC [N.G.]. Funding for open access charge: Wellcome Trust.

## SUPPLEMENTARY DATA

Supplementary Data are available at NAR Online, including supplementary references [1–4].

SUPPLEMENTARY DATA
