# Supplementary material for: Recombination hotspots attenuate the coupled ATPase and translocase activities of an AddAB-type helicase–nuclease
Source: Nucleic Acids Res. 2014 Mar 15;42(9):5633–43. doi: 10.1093/nar/gku188 (PMC4027173; doi:10.1093/nar/gku188)
Supplement: SUPPLEMENTARY DATA [file supp_42_9_5633__index.html]

Recombination hotspots attenuate the coupled ATPase and translocase activities of an AddAB-type helicase–nuclease — SUPPLEMENTARY DATA 

# Recombination hotspots attenuate the coupled ATPase and translocase activities of an AddAB-type helicase–nuclease

## SUPPLEMENTARY DATA

**Files in this Data Supplement:**

- Supplemental Figures
