# Supplementary material for: Recombination hotspots attenuate the coupled ATPase and translocase activities of an AddAB-type helicase–nuclease
Source: Nucleic Acids Res. 2014 Mar 15;42(9):5633–43. doi: 10.1093/nar/gku188 (PMC4027173; doi:10.1093/nar/gku188)
Supplement: SUPPLEMENTARY DATA [file supp_gku188_nar-03542-d-2013-File008.pdf]

Recombination hotspots attenuate the coupled ATPase and translocase activities of an AddAB-type helicase-nuclease.

Neville S. Gilhooly and Mark S. Dillingham*

DNA-Protein Interactions Unit, Department of Biochemistry, School of Medical Sciences, University of Bristol, University Walk, Bristol, BS8 1TD, UK
*To whom correspondence should be addressed. Tel: +44 117 3312159; Fax: +44 117 3312168; Email: mark.dillingham@bristol.ac.uk

SUPPLEMENTARY MATERIAL

Supplementary text

Materials and Methods

Discussion 1: In silico modelling of triplex displacement by AddAB

Discussion 2: Correct substrate design allows for model discrimination.

Discussion 3.1: Assessing accuracy of semi-quantitative fitting of triplex displacement data.

Discussion 3.2: Assessing the precision of semi-quantitative fitting of triplex displacement data.

Discussion 4: Phosphate release assay controls.

Supplementary tables 1-4

Supplementary figures

Figure S1 – Schematic of triplex displacment models that result in a delay of AddAB at the triplex.

Figure S2 – *A priori* lag times associated with directional translocation for AddAB enzymes that undergo a rate change, a pause, or both a rate change and a pause at Chi.

Figure S3 – Changing the distance between Chi and the triplex can in principle allow the determination of the post-Chi translocation rate using a triplex displacement assay.

Figure S4 – Assessing the accuracy of semi-quantitative fitting routines to modelled triplex displacment data.

Figure S5 – Examining the cross-correlation between parameters in Equation 1.

Figure S6 - The translocation rate of AddAB reproducibly decreases following Chi recognition.

Figure S7 – Heparin is an effective trap for AddAB and the phosphate mop acitivty is not significantly active during the time scale of experiments.

Figure S8 – The translocation rate determined from the ATPase rate change on Chi-containing DNA agrees with end-point analysis on Chi-free DNA.

Figure S9 – The amplitudes of phosphate release using Chi-containing or Chi-free substrates are similar.

SUPPLEMENTARY TEXT

Materials and Methods:

The following table (Table S1) shows the primer combinations and parent plasmids used to make DNA substrates used in this study.

Forward primer	Reverse Primer	Parent plasmid	Figure in main text	Total length (b.p)
bioNGF2<br>bio-CGATTATTATATATACCATTTCAATGTTC	NGR3<br>TTTTATTGTATTCATCTTTGCAATTATAC	pSP73-JY0 TFO	1	1392
bioNGF2	NGR4<br>ATCGAAGAAAGTGCATAATGTAGGC	pSP73-JY0 TFO	1	2412
bioNGF2	NGRB<br>GGTGGGTTTGCCAACAAATG	pSP73-JY0 TFO	1	3022
bioNGF2	NGRC<br>CTACCAGTTCGGGACTAGCTTAC	pSP73-JY0 TFO	1	3553
bioNGF2	NGR2<br>GATACAAATAACTCTTTGTATCATAGTATTCTC	pSP73-JY0 TFO	3	2824
bioNGF2	NGR2	pSP73-JY1 TFO	3	2824
bioNGF2	NGR2	pSP73-JY2 TFO	3	2824
bioNGF2	NGR2	pSP73-JY10 TFO	3	2824
bioNGF2	NGRB	pSP73-JY10 TFO del	5	2008
bioNGF2	NGRB	pSP73-JY10 TFO	5	3020
bioNGF2	NGRB	pSP73-JY10 TFO i2	5	4011

bioNGF2	NGRB	pSP73-JY10 TFO i1	5	4811
bioNGF2	NGR7<br>TCAGTCTTGCAGAAGGACT G	pSP73-JY10	6	400
bioNGF2	NGR8<br>TGATAAAAGCCAACTTACT GATG	pSP73-JY10	6	600
bioNGF2	NGR9<br>TTCAAAAGAGCTGAATGCT AC	pSP73-JY10	6	900
bioNGF1<br>bio- GGTAAAACGGCTCGATG ACTTC	NGR7	pSP73-JY10	6	1231
bioNGF4<br>bio- CGATATGAACATAGTCG TTAACTTGC	NGR3	pSP73-JY0-BbvCI-Superchi - Forward	7	1957
bioNGF4	NGR4	pSP73-JY0-BbvCI-Superchi - Forward	7	2212
bioNGF4	NGR5<br>CGCGGAACCCCTATTTG	pSP73-JY0-BbvCI-Superchi - Forward	7	2832
bioNGF4	NGR3	pSP73-JY0-BbvCI-Superchi - Reverse	7	1957
bioNGF4	NGR4	pSP73-JY0-BbvCI-Superchi - Reverse	7	2212
bioNGF4	NGR5	pSP73-JY0-BbvCI-Superchi - Reverse	7	2832

Discussion 1: In silico modelling of Triplex displacement by AddAB

Triplex displacement models were constructed in Berkeley Madonna (Robert I. Macey & George F. Oster).Two models were considered to account for the delay of AddAB arriving at the triplex, a single pause at Chi or a translocation rate decrease following Chi recognition. Figure S1 shows a schematic of both of these models. Both models are “n step” sequential translocation models where each state in the model corresponds to one base pair translocated. The reason for choosing this kinetic step size was to avoid potential artefacts associated with the spatio-temporal distribution of enzymes along DNA that possess an unrealistic kinetic step size.

In both models enzymes initiate translocation from “A1” and take a series of irreversible unimolecular steps (n), governed by the unimolecular rate constant “*k*”, along a linear 1D DNA lattice until Chi is reached. Two fast rate constants “*kchi*” and “*knotchi*” (~5 fold that of the translocation rate) are used to split the translocating population in a manner that is predictable and does not affect the underlying distribution of the translocating population. Enzymes that “recognise” Chi either undergo a single pause or change their translocation rate. The dwell at the position of Chi is determined by the rate constant “*kpause*”. Both populations of enzyme then take “m” steps after Chi to reach the triplex, the rate of which is determined by the value of “*k*” for enzymes that pause and “*k2*” for enzymes that change rate. When enzymes reach the triplex they displace it with a rate governed by “*ktriplex*”.

The Berkeley Madonna scripts describing the system of coupled differential equations for these models are available from the corresponding authors upon request.

Discussion 2: Correct substrate design allows for model discrimination.

As alluded to in the main text, multiple models are capable of explaining the delay in the arrival of AddAB at the triplex. A simple thought experiment can illustrate how correct substrate design can distinguish between all of these models. The time it takes for AddAB to reach any position on a hypothetical DNA substrate that has a Chi locus 1000 b.p. away from the free DNA end is considered. AddAB enzymes translocate at a rate of 2000 b.p.s^{-1} and so the time taken to reach any position on the substrate, if Chi is never recognised, is given by the distance divided by the rate (black line in Figure S2). Enzymes that do recognise Chi (AddAB*) might either undergo a stochastic or non-exponentially distributed pause at Chi. Alternatively, AddAB* might decrease rate after Chi or perhaps both pause at Chi and change rate. One can calculate *a priori* the average time it takes to translocate a certain distance for each of the AddAB and AddAB* populations. These values are plotted as a function of DNA length, up to 10 kb.p. (Figure S2). Note that the arrival times plotted for AddAB and AddAB* are potentially equivalent to the T1 and T2 parameters that can be derived experimentally. The black line in Figure S2 corresponds to values of T1 and the coloured lines correspond to values of T2 within different models. Both the pause and rate change models yield dramatically different dependencies of T2 on the distance translocated. If AddAB undergoes a change of rate after Chi, the value of T2 starts to diverge beyond the position of Chi yielding a line with a steeper gradient that intersects with the T1 line at the position of Chi (Figure S2 – compare red and black lines). However,

if AddAB pauses at Chi, T2 values form a parallel line (equivalent gradient) with respect to T1 which is offset on the y axis. This offset is precisely equivalent to the pause duration (Figure S2 – compare blue and black lines). The mixed model shows both behaviours, the T2 line both diverges from the T1 line and is also offset. The value of this offset at the position of Chi is equivalent to the pause duration.

Discussion 3.1: Assessing accuracy of semi-quantitative fitting of triplex displacement data.

The thought experiment described above shows that changing the distance between Chi and the triplex and measuring the values T1 and T2 can potentially provide a simple way to experimentally distinguish between the models proposed above, all of which result in the delayed triplex displacement by AddAB* that we observe. However, this relies on a semi-quantitative fitting approach being able to return the values of T1 and T2 reliably. Therefore, as a final confirmation that this approach was viable, triplex displacement curves were simulated for just such an experiment, and then a simple two-exponential fitting routine was used to back-extract the inputted (model) values. For example, triplex displacement with DNA substrates with a variable distance between Chi and the triplex were simulated within the framework of the combined rate change and pause model and fit to Equation 1 in order to determine if Equation 1 returns correct values for the modelled translocation rates of AddAB and AddAB* (Figures S3B and S3C). Equation 1 accurately determines the translocation rates of AddAB and AddAB* as 1877 b.p.s^{-1} and 1467 b.p.s^{-1} (<2% error); the model values are 1880 b.p.s^{-1} and 1440 b.p.s^{-1}. The pause duration calculated from the difference between the values of T2 and T1 at 353 b.p. is 0.16 seconds. This differs from the modelled value (0.2 seconds) by 20%, probably reflecting inaccuracies in the extrapolated values of the y-axis intercepts which are used to calculate the pause duration. Nevertheless, the fact that the linear fits to values of T1 and T2, extracted from the modelled data using Equation 1, do not intersect at the position of Chi shows that this method could be used to provide evidence for a pause at Chi from experimental data.

Discussion 3.2: Assessing the precision of semi-quantitative fitting of triplex displacement data.

To further confirm the validity of the approach taken to fit triplex displacement data, Monte Carlo (MC) analysis was performed. A single triplex displacement trace was simulated using the rate change model described above and shown in Figure S4. The pre- and post-Chi translocation rates were set to 1880 and 1450 b.p.s^{-1} with 50% Chi recognition and the simulated data was fit with Equation 1. The standard error usually reported in statistical software packages is an asymptotic standard error which requires a linear fitting function and the absence of correlation between parameters; it is thus not suitable for the equations one can derive when encountering a kinetic problem (1). MC analysis is a preferred method of choice for establishing confidence intervals of parameters obtained from a NLLS fitting routine and the degree of their cross-correlation, if any.

MC analysis works on the following principles, formalised by Press et al (2): a statistical universe of data sets exists based on an underlying model with parameters a_{true} that are hidden to the

observer. A measured data set, $D_{o,}$ contains the true parameter values, $a_{true,}$ but also experimental error and thus yields a set of parameters derived from NLLS termed, a_o. D_o is therefore not a unique representation of the true parameters, a_{true}. There are an infinite number of experiments, $D_{i,}$ that could be performed, each yielding their own values of a (a_i); a is therefore described by a probability distribution. One is interested in estimating the distribution of the deviation of a_i from a_{true} when only D_0 is available. The assumption of MC analysis is that the shape of the distribution of $a_i – a_o$ in the statistical universe is equivalent to $a_i – a_{true}$ in the real world. Synthetic data sets are generated that have the same statistical relationship to a_o and a_{true}. Therefore when parameters are obtained by NLLS from these data sets, a value for (a_i – ao) is yielded. If enough data sets are simulated the probability distribution for $a_i – a_{true}$ is empirically populated.

MC analysis was performed in 4 steps (3):

1: Data (in this case the stepping model) is fitted to a mathematical model (Equation 1) to derive parameter values and then an “ideal model data set” is generated from Equation 1 using the best fit values for parameters.

2: Gaussian noise is added to the ideal data set with a standard deviation equivalent to the standard deviation of the residuals of the fit (Sy,x in GraphPad Prism).

3: The ideal data set is generated with noise and fit to Equation 1 and fitted parameters tabulated.

4: Step 3 was repeated 230 times to get a large enough sample size to generate meaningful histograms.

Whilst a simulated model has no error associated with it, we are applying a semi quantitative fit which introduces its own error as it lacks the mathematical features necessary to describe all parts of the time course of triplex displacement. We are therefore interested in determining the range over which parameters can take on values and if a unique best fit to the modelled data can be obtained. This can provide confidence that Equation 1 is an appropriate semi-quantitative fit to the biphasic triplex displacement reported in the main text. The histograms generated from MC analysis were fitted to a Gaussian distribution to determine the mean parameter value and its standard deviation shown in Figure S4. All parameters are determined with low standard deviations. Importantly, In the case for the two lag times, T1 and T2 there is not sufficient variation to give rise to a Gaussian distribution showing these two values, in particular, are extremely robust. The absence of any bimodal distributions suggests that a unique fit has been found and that the NLLS routine was not stuck in any local minima. In support of this, Figure S3 shows that Equation 1 yields the correct translocation rate for both model Chi-modified and unmodified enzymes.

Perhaps more informative is the assessment of correlation between parameters. Figure S5 shows

cross-correlation plots of the parameters in Equation 1. Parameters that show correlation are A2:A1, *k*1:A1 and *k*1:A2. The main source of error in the fit seems to be fitting of the first phase. If the

rate constant for the first phase is too high the amplitude, A1, must decrease in order to maintain a good fit. This has the obvious knock on effect that A2 increases to compensate for the loss in A1. A2 and A1 are therefore highly anti-correlated, however the range of values taken by both amplitudes is within ± 2 % which is tolerable when measuring amplitudes, as in Figure 2C, where conclusions were made about amplitude values at least five times greater than this error (main text). Both lag times are essentially invariant in the fit showing that these are the most robustly determined parameters in the fit. Of course the noise inherent in experimental data will lead to larger errors on fitted parameters, but this does not affect the conclusions reached in the main text.

Discussion 4: Phosphate release assay controls

The efficacy of using heparin as a trap for ATPase measurements was examined. Heparin was included with AddAB and DNA before rapid mixing against ATP in a stopped-flow device. No ATPase activity was observed (Figure S7) indicating that heparin effectively competed against DNA ends for binding to AddAB, and that AddAB bound to heparin does not turnover ATP at an appreciable level. Omitting heparin results in biphasic ATPase activity, however when heparin is added to the syringe containing ATP, the second phase is greatly reduced allowing resolution of the burst amplitude of ATP hydrolysis that is associated with translocation from one DNA end to the other. Regardless of the presence or absence of heparin the rapid phases have very similar rates and amplitudes, suggesting minimal perturbation of (1st phase) AddAB translocation activity unlike what has been observed in some other systems (4).

Further controls were done in which 1 µM Pi was added to the DNA containing syringe instead of AddB and then rapidly mixed against an equal volume of a solution containing ATP, heparin and mop components (see materials and methods in main text) to determine the extent of mop activity. No significant activty was observed over 5 seconds (Figure S7). ATPase activity was also dependent upon the addition of AddAB.

Table S2 – Best fit parameters obtained by fitting Equation 1 to triplex displacement data in Figure 1 (main text)**.** Black values represent wild-type and purple indicates AddAB^{F210A}.

**Distance to TFO (b.p.)**	**1319**	**2339**	**2949**	**3480**
**T1 (s)**	0.7703±0.001104<br>0.7509±0.001085	1.354±0.00179<br>1.307±0.001079	1.61±0.001925<br>1.621±0.001468	1.951±0.001922<br>1.894±0.001558
**A1 (% of max)**	92.9 ±0.2772	90.27±0.4984	90.31±0.4113	90.21±0.417

	90.46±0.4008	91.69±0.2539	91.81±0.2591	89.18±0.3353
***k*1 (s^{-1})**	15.11±0.4055<br>13.42±0.3572	7.594±0.208<br>10.29±0.1948	7.988±0.2225<br>9.172±0.2038	7.645±0.2042<br>9.456±0.2389
**T2 (s)**	1.601±0.1745<br>1.206±0.256	2.006±0.08021<br>2.072±0.09424	2.415±0.08337<br>2.669±0.08312	2.767±0.0817<br>2.707±0.0886
**A2 (% of max)**	7.1±1.814<br>9.54±2.738	9.73±0.7735<br>8.31±1.098	9.69±0.9629<br>8.19±1.053	9.79±1.308<br>10.82±1.994
***k*2 (s^{-1})**	0.3896±0.1805<br>0.1854±0.1491	0.8187±0.1401<br>0.5278±0.1408	0.8659±0.2047<br>0.8372±0.2514	0.862±0.2367<br>0.6137±0.211

Table S3 – Best fit parameters obtained by fitting Equation 1 to triplex displacement data in Figure 2 (main text). Black values represent wild-type and purple indicates AddAB^{F210A}.

**Chi**	**0**	**1**	**2**	**3**
**T1 (s)**	1.504±0.001504<br>1.472±0.001304	1.525±0.00232<br>1.483±0.001834	1.53±0.002427<br>1.493±0.001301	1.542±0.003172<br>1.511±0.001549
***k*1 (s^{-1})**	8.314±0.2070<br>8.447±0.1769	7.602±0.269<br>10.86±0.3880	7.277±0.2588<br>9.928±0.2415	5.301±0.2451<br>8.829±0.2271
**A1 (% of max)**	89.26±0.4359<br>90.88±0.3569	73.39±0.5161<br>88.82±0.5366	61.60±0.4467<br>89.52±0.3911	57.07±0.7481<br>85.79±0.3982
**T2 (s)**	2.145±0.04896<br>2.141±0.05733	2.229±0.01714<br>2.084±0.6988	2.207±0.007504<br>2.087±0.04305	2.163±0.006528<br>2.162±0.03073
***k*2 (s^{-1})**	1.2±0.141<br>0.9737±0.1318	1.786±0.09058<br>1.043±0.1727	2.496±0.06827<br>1.332±0.1393	2.932±0.07518<br>1.413±0.1122
**A2 (% of max)**	10.74±0.5543<br>9.12±0.5405	26.61±0.5841<br>11.18±0.7838	38.40±0.4755<br>10.48±0.4766	42.93±0.7621<br>14.21±0.4801

Table S4 – Best fit parameters obtained by fitting Equation 1 to both sets of triplex displacement data in Figures 4 (main text) and S6. Colour codes correspond to the experimental traces in the main text (Figure 4) and Figure S6. Note that blue and green amplitudes do not add up to 100% due to a y-axis offset that is described in the main text.

**Distance from DNA end to TFO (b.p.)**	1980	2993	3983	4783	
**T1 (s)**	1.048±0.004866	1.539±0.003902	2.155±0.008267	2.698±0.005907	Figure 4
**A1 (% of max)**	70.45±1.48	66.0±0.1356	45.23±5.176	64.23±1.95	
***k*1 (s^{-1})**	3.245±0.08164	2.419±0.08637	2.452±0.4579	1.344±0.05703	
**T2 (s)**	1.355±0.0061	2.069±0.005757	2.631±0.01043	3.345±0.01023	
**A2 (% of max)**	29.55±1.434	34.0±1.327	43.17±5.249	25.41±1.64	
***k*2 (s^{-1})**	14.2±2.053	5.417±0.3795	1.737±0.05104	2.904±0.2644	
**T1 (s)**	1.05±0.005669	1.532±0.004614	2.158±0.005764	2.665±0.004461	Figure S6
**A1 (% of max)**	49.82±7.99	48.85±2.819	45.77±4.075	61.95±1.941	
***k*1 (s^{-1})**	5.951±1.481	4.157±0.4903	2.455±0.3680	1.468±0.0666	
**T2 (s)**	1.271±0.007374	1.915±0.006550	2.58±0.007963	3.218±0.007279	
**A2 (% of max)**	50.18±8.005	51.15±2.822	41.34±4.136	30.72±1.74	
***k*2 (s^{-1})**	3.54±0.09808	2.395±0.04546	1.658±0.03267	1.519±0.1753	

SUPPLEMENTARY FIGURES

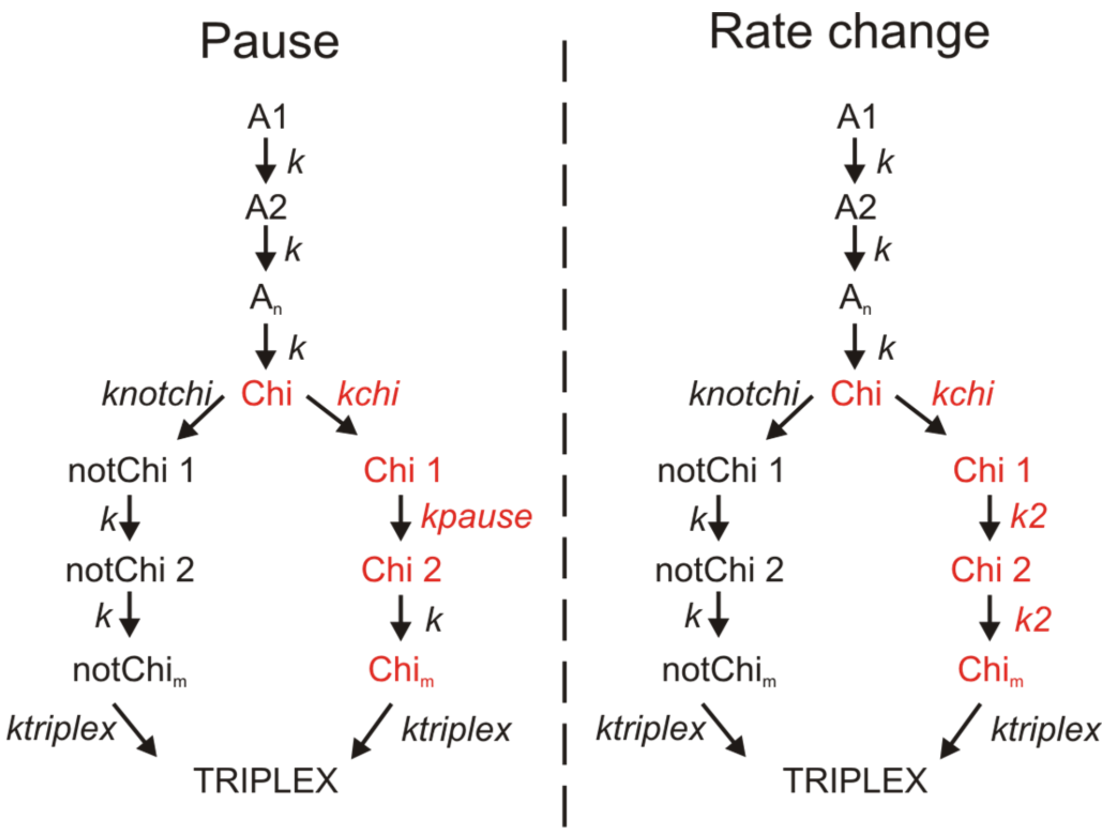

Figure S1 – Schematic of triplex displacment models that result in a delay of AddAB at the triplex.

Details of the parameter values can be found in the main text, red indicates Chi-specific parameters.

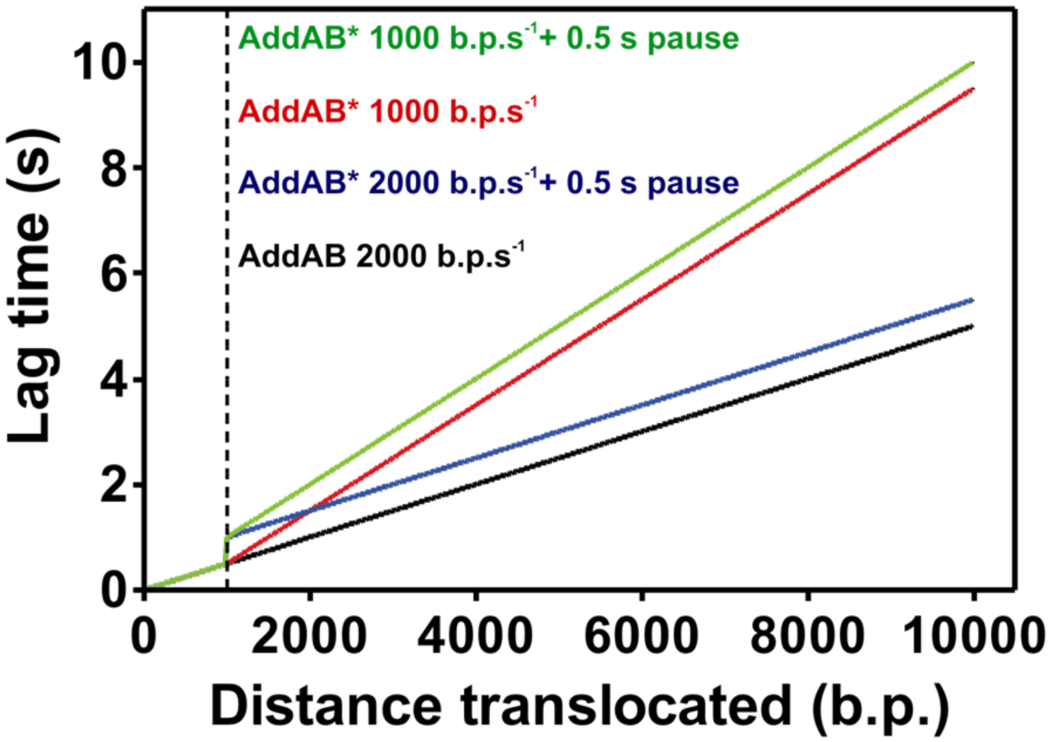

Figure S2 – *A priori* lag times associated with directional translocation for AddAB enzymes that undergo a rate change, a pause, or both a rate change and a pause at Chi.

The lag time associated with a particular distance translocated was simulated for AddAB enzymes that translocate at 2000 b.p.s^{-1} (black line). When these enzymes reach the Chi locus, enzymes are converted to AddAB* giving rise to AddAB*-specific lag times. Three models for the behaviours of AddAB* were considered; a pause of 0.5 seconds (blue line), a rate decrease to 1000 b.p.s^{-1} (red line) or both a rate decrease and a pause (green line). The position of the Chi locus is indicated as a black dotted line.

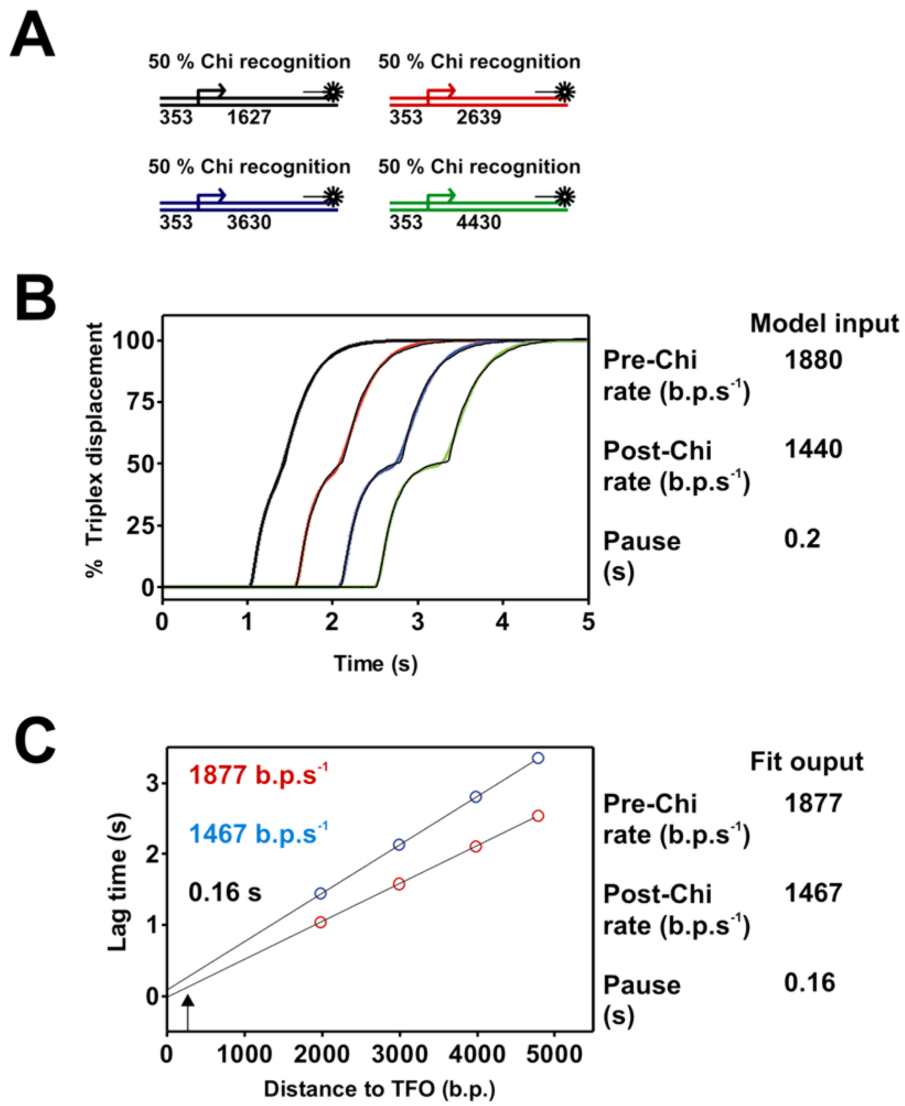

Figure S3 - Changing the distance between Chi and the triplex can in principle allow the determination of the post-Chi translocation rate using a triplex displacement assay.

(A) Schematics of *in silico* substrates used for simulations. The distance to Chi is 353 b.p. in all cases and the distance between Chi is varied from 1627 (black) to 4430 b.p. (green). (B) Simulation of the combined rate change and pause model using the same distances as in (A). The translocation rate for AddAB is set at 1880 b.p.s^{-1} and and it is assumed 50% of enzymes recognise Chi. The post-Chi translocation rate is set to 1440 b.p.s^{-1} and enzymes that recognise Chi pause on average for 0.2 seconds. Triplexes are displaced with a first order rate constant of 6 s^{-1} by both AddAB and AddAB* The black lines are fits to the modelled data using Equation 1 to extract the values for the T1 and T2 lag times. (C) Plot of the T1 (red circles) and T2 (blue circles) lag times as a function of distance from the DNA end to the triplex. Linear fits to the data yield accurate translocation rates for AddAB (red) and AddAB* (blue). The arrow indicates the position of Chi. Note that the linear fits do not intersect here, and the y-axis offset between the fitted lines equates to the pause duration (0.16 s).

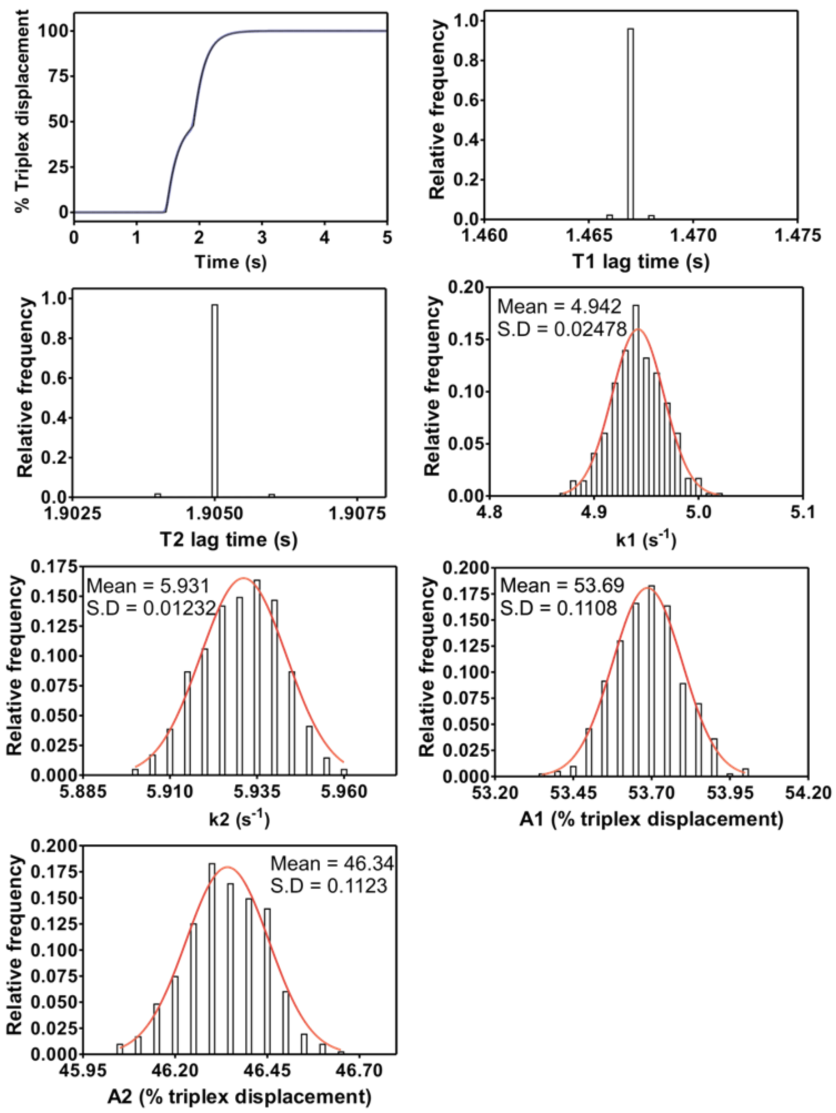

Figure S4 – Assessing the accuracy of semi-quantitative fitting routines to modelled triplex displacment data

(Top left) Simulation of rate change model (blue line) where pre- and post-Chi translocation rates were set to 1880 and 1450 b.p.s^{-1} with 50% Chi recognition. The distance to the Chi locus is 134 b.p and the distance from Chi to the triplex is 2640 b.p. The black line is a fit using Equation 1. (Rest) Monte Carlo estimation of fitted parameters determined from 230 synthetic data sets. The red line is a Gaussian fit to histograms generated from Monte Carlo analysis, with mean and standard deviation reported in each panel. The error in determining T1 and T2 is not sufficient to give rise to a Gaussian distribution and each of these parameters is essentially captured as a single value.

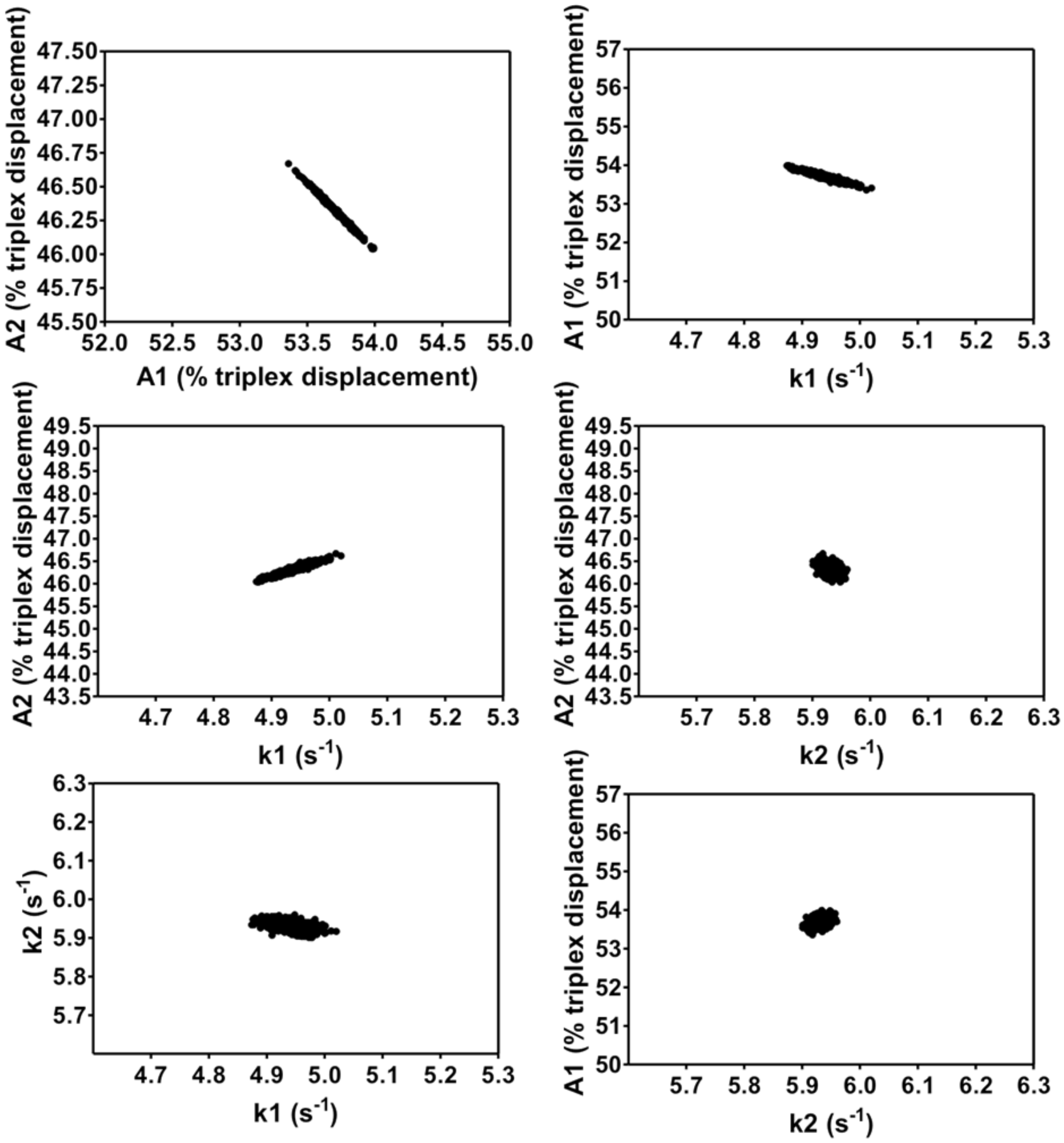

Figure S5 – Examining the cross-correlation between parameters in Equation 1

Parameters that show correlation that was judged by eye are A2:A1, *k*1:A1 and *k*1:A2.

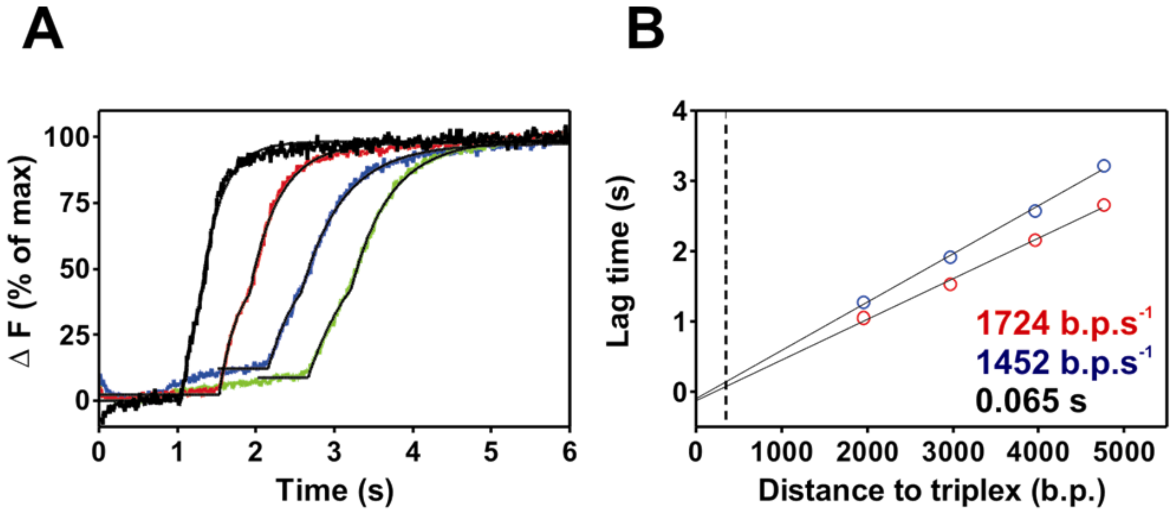

Figure S6 - The translocation rate of AddAB reproducibly decreases following Chi recognition.

An independent triplex displacement experiment to that displayed in the main text (Figure 4) was performed in order to confirm the reproducibility of the translocation rate decrease after Chi.

(A) Triplex displacement experiment with wild-type AddAB on Chi-containing DNA with variable distances between Chi and the triplex binding site. The distances and colour coding are identical to those in substrates used for modelling (Figure S3). DNA molecules (2 nM) blocked on one end by a biotin:streptavidin complex were prebound by AddAB enzymes (10 nM) for 2 minutes at 37 ^{o}C before mixing against an equal volume of ATP (1 mM) and AddA^{K36A}B (200 nM). Data are the average of at least 3 transients and normalised to the fluorescence endpoint. Black lines indicate fits to the data using Equation 1. The blue and green traces are only fit from 1.5 and 2 seconds onwards respectively (see main text for discussion) (B) Plot of the the first and second phase lag times, T1 (red) and T2 (blue) as a function of DNA length. Linear fits to these data yield values for the pre- and post-Chi translocation rate. The black dotted line is the position of the Chi locus.

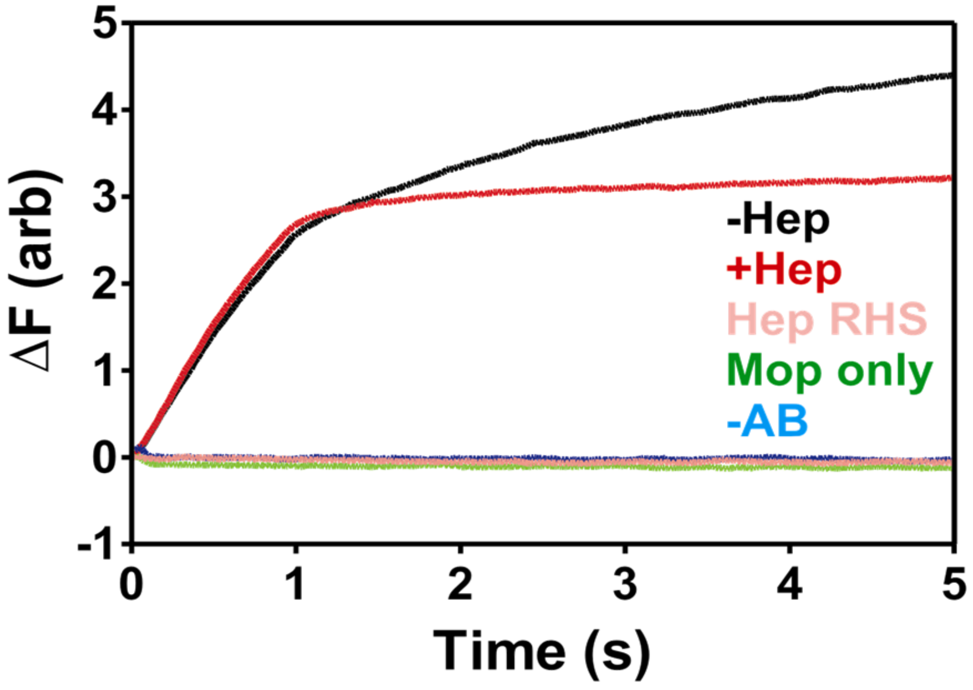

Figure S7 - Heparin is an effective trap for AddAB and the phosphate mop activity is not significantly active during the time scale of experiments.

Phosphate release experiments performed on F4R3 Superchi reverse DNA. DNA substrates (0.2 nM) were pre incubated with AddAB enzymes (2 nM) for 2 minutes at 37°C before mixing against an equal volume of ATP (1 mM) and heparin (1 mg.mL^{-1}) (red trace). Heparin was omited (black) or incubated in the same syringe as DNA before mixing against ATP and AddB (pink). 1 µM Pi was added and AddAB was ommited (green) to determine the activity of the mop over 5 seconds, this data was normalised to start at 0. AddAB was ommited (blue) showing ATPase actiity is AddAB dependent.

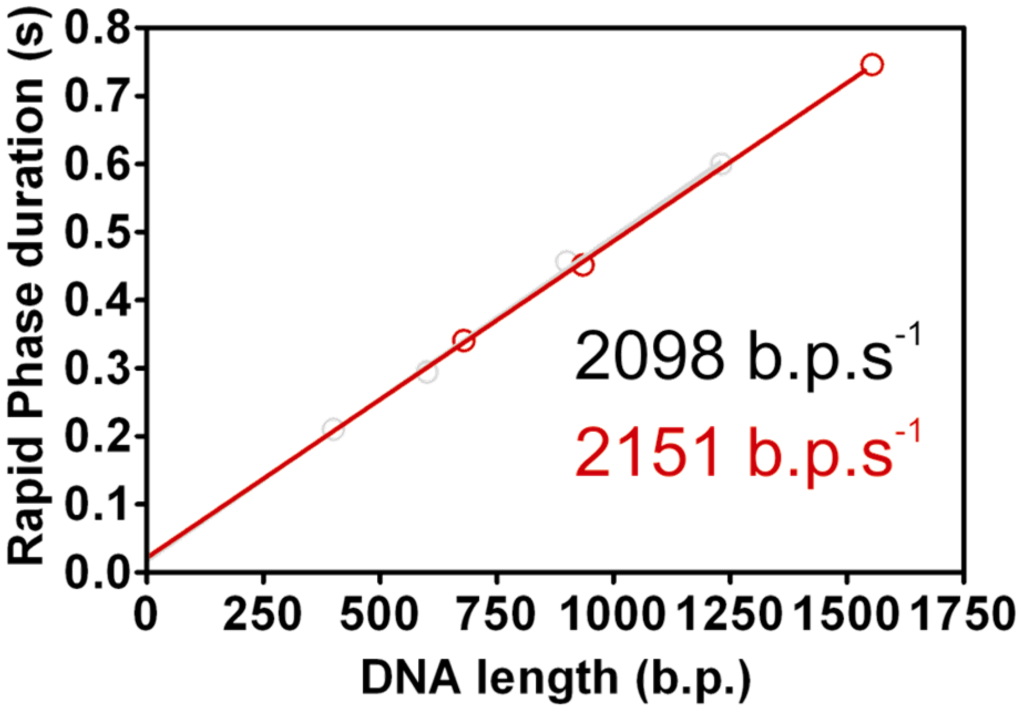

Figure S8 – The translocation rate determined from the ATPase rate change on Chi-containing DNA agrees with end-point analysis on Chi-free DNA.

The red points indicate the time at which the Chi-dependent decrease in ATPase activity occurs in the data shown in Figure 6 (main text). The red line is a linear fit to the data, the gradient of which yields the translocation rate of AddAB. The greyed out data is that of Figure 5C in the main text. Note the highly overlapping lines and similar translocation rates obtained.

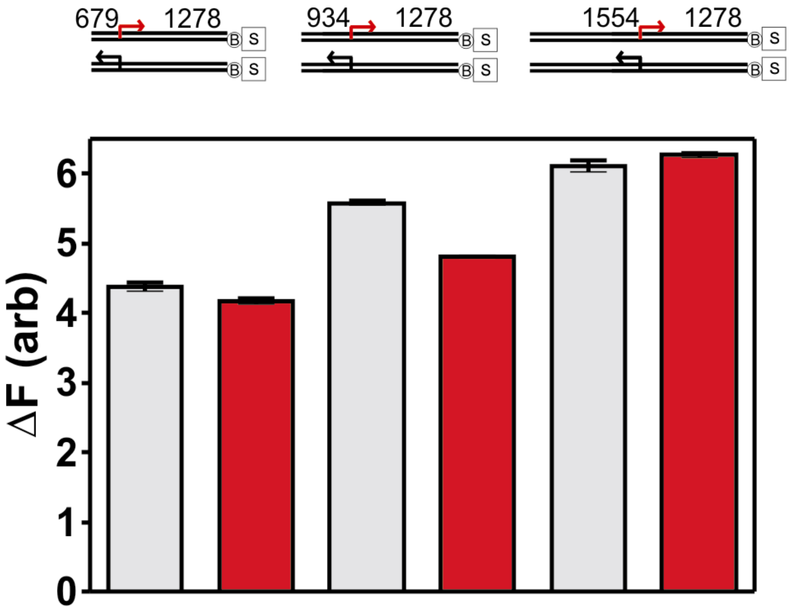

Figure S9 – The amplitudes of phosphate release using Chi-containing or Chi-free substrates are similar.

Amplitudes of the rapid phase of ATPase activity on reverse (grey bars) and forward (red bars) 10X Chi substrates. A schematic of the substrates is shown above each pair of bars. Error bars represent the S.E.M. determined from two experiments.

Supplementary References

1. Herman, P. and Lee, J.C. (2011) The advantage of global fitting of data involving complex linked reactions. *Methods Mol Biol*, **796**, 399-421.
2. Press WH, T.S., Vetterling WT, Flannery BP (1992) Numerical Recipes in C: The Art of Scientific Computing, 2nd ed. . *Cambridge: Cambridge University Press*, **pp 498, 521–525**.
3. Motulsky, H. and Christopoulos, A. (2004) *Fitting Models to Biological Data using Linear and Nonlinear Regression. A Practical Guide to Curve Fitting.*
4. Fischer, C.J., Maluf, N.K. and Lohman, T.M. (2004) Mechanism of ATP-dependent translocation of E.coli UvrD monomers along single-stranded DNA. *J Mol Biol*, **344**, 1287-1309.
